# Theoretical Analysis of Electronic, Vibrational Properties, and Noncovalent Interactions in Methylxanthines: Monomers to Antiparallel Dimers

**DOI:** 10.1021/acsomega.6c02053

**Published:** 2026-08-10

**Authors:** Raúl Mendoza-Báez, Dolores Garcia-Toral, Alexandra Deriabina, María Patricia Sánchez Gutiérrez, Víctor M. Vázquez-Báez, Akari Narayama Sosa Camposeco, Alexandra Bustamante Camacho, Gregorio Hernández Cocoletzi, Juan Francisco Rivas-Silva

**Affiliations:** † Departamento de Química, Centro de Investigación y de Estudios Avanzados del IPN (Cinvestav), Avenida IPN 2508, Col. San Pedro Zacatenco, Ciudad de México 07360, México; ‡ Facultad de Ingeniería Química, Benemérita Universidad Autónoma de Puebla, Av. San Claudio y 18 Sur S/N, San Manuel, Puebla 72570, México; § Facultad de Ciencias Físico Matemáticas, Benemérita Universidad Autónoma de Puebla, Puebla 72570, México; ∥ Facultad de Ingeniería, Benemérita Universidad Autónoma de Puebla, Puebla 72570, México; ⊥ Instituto de Física “Ing. Luis Rivera Terrazas”, Benemérita Universidad Autónoma de Puebla, Av. San Claudio y Blvd. 18 Sur, Col. San Manuel, Ciudad Universitaria, Puebla, Pue. 72570, México

## Abstract

This study provides a comprehensive characterization of methylxanthines in monomeric and antiparallel dimeric configurations using DFT-D3 and SAPT0 analysis. Our results elucidate a synergistic interplay between hydrogen bonding and π–π stacking, where London dispersion accounts for over 50% of the total attractive forces. The wide HOMO–LUMO gaps (7.2–9.4 eV) obtained with M06–2X and ωB97X correlate with minimal induction contributions (E_ind_ < 9.5%) and negligible charge transfer, confirming the noncovalent nature of the assembly. A detailed thermochemical analysis confirms the spontaneity of dimerization through calculated Gibbs free energies, revealing a strong dependence on the chosen density functional. Furthermore, color-coded NCI-RDG plots provide an unambiguous mapping of attractive and steric forces, demonstrating that while dimerization preserves monomeric reactivity, it drives a stable supramolecular assembly. These insights provide a fundamental starting point for understanding the intrinsic driving forces of methylxanthines, potentially aiding in the future rational design of methylxanthine-based cocrystals and supramolecular materials.

## Graphical abstract

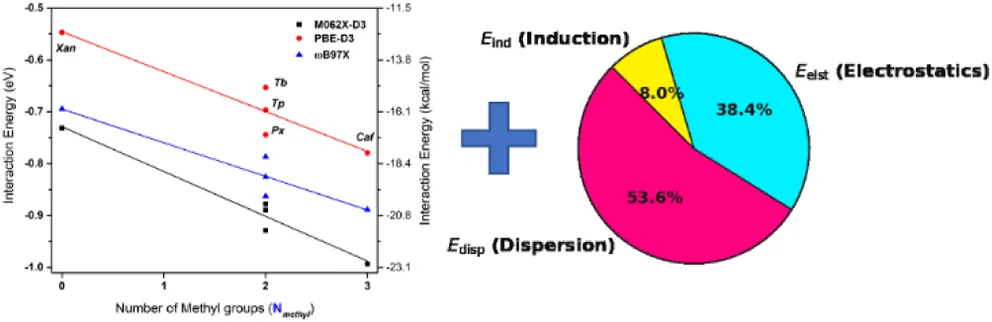

## 1 Introduction

Methylxanthines, belonging to the purine group, are a class of naturally occurring alkaloids present in, e.g., coffee, tea, cocoa, and yerba mate, and they play important physiological.^1–4^ When methylxanthines are absorbed, they penetrate the central nervous system, causing psychostimulant effects due to their antagonistic action on adenosine receptors in the brain.^5–10^ The main molecules within the methylxanthine family are caffeine (1,3,7-trimethylxanthine), theobromine (3,7-dimethylxanthine), theophylline (1,3-dimethylxanthine), and paraxanthine (1,7-dimethylxanthine), which are distinguished by the number and position of methylation (Figure 1). Caffeine is undoubtedly the most prominent example, as it is the most widely consumed.^11–13^ Specific methylation at the *xanthine*-scaffold results in each methylxanthine exhibiting a characteristic physiological activity and metabolic behavior, as recently described by Jiang et al.^14^ Thus, methylxanthines are of interest in the pharmaceutical industry for drug development, but they have also shown potential in other areas such as food technology, catalysis, and self-assembled materials.^15–23^ Therefore, the biosynthetic mechanisms of xanthine derivatives are currently being studied in depth.^14,24–26^

**Figure 1:**
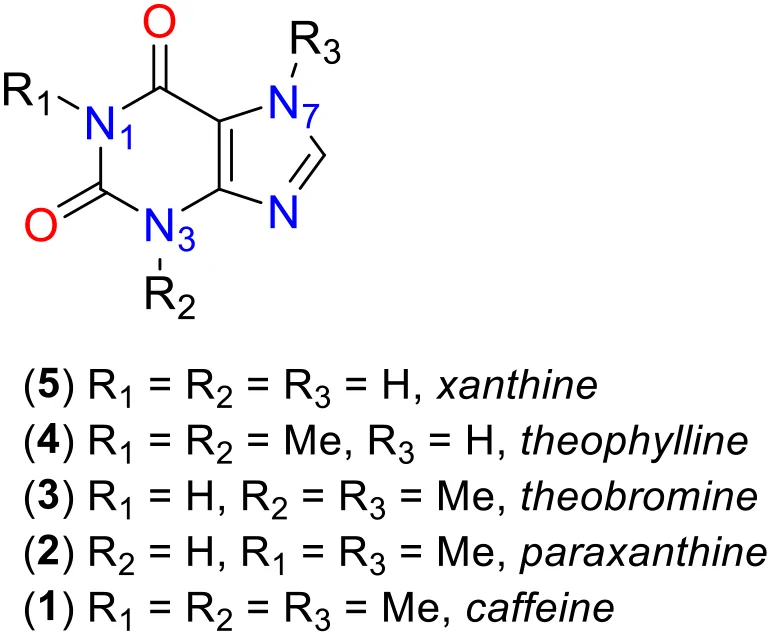
Molecular structure of the (methyl)­xanthines studied in this work.

Although various methylxanthines have been evaluated through *in vivo*, *in vitro,* and *in silico* experiments for pharmacological applicability,^27–30^ they have also been studied theoretically to understand their properties at the molecular level. Callahan et al.^31^ used R2PI and IR-UV spectroscopy techniques and DFT-D frequency calculations to study the intermolecular interactions governing the formation of some methylxanthine dimers, finding that increasing the number of methylated sites favored π–π stacking over hydrogen bonding. Later, Latosinska et al.^32^ analyzed the topology of the interactions in caffeine, theobromine, and theophylline in the solid state by examining the local environment of the nitrogen nucleus using ^1^H–^14^N NMR-NQR double resonance, with the goal of associating the nature of these interactions with the specific biological processes they undergo. Interestingly, they also found that the methyl groups on xanthine inhibit the participation of nitrogen atoms in strong hydrogen bonds, causing π–π stacking to be the dominant interaction, consistent with what was previously described by Callahan et al.^31^ On the other hand, Gibson and Fowler verified the aromaticity of xanthine, caffeine, theobromine, theophylline and paraxanthine since these molecules presented delocalized π–electron ring-currents delocalized mainly around the imidazole moiety, based on *Coupled Hartree–Fock* (CHF) and DFT calculations.^33^ Additional vibrational and spectroscopic studies have been reported for some methylxanthines (mainly caffeine, theobromine and theophylline); however, these focus exclusively on monomers.^34–37^ Recently, solvent-promoted noncovalent interactions in (methyl)­xanthines in solution have also been investigated.^38–40^ All of the above support the interest in understanding in depth the mechanisms underlying the molecular assembly of methylxanthines, since this would allow us to better elucidate the nature of some recognition processes in physiological systems.

As shown above, the supramolecular chemistry of (methyl)­xanthines is mainly based on hydrogen bonding and π–stacking interactions. Stacking interactions are vital in biological systems such as DNA and proteins and to occur primarily in aromatic molecules.^41–44^ These interactions also play a fundamental role in the solid-state chemistry of (methyl)­xanthines. S. Gutierrez et al.,^45^ studied the structural properties and *stacking* characteristics of anhydrous caffeine crystals using quantum mechanical methods, observing a global minimum dimer with zero dipole moment, in which the individual dipole vectors were antiparallel. On the other hand, the enthalpy of sublimation and the crystalline data of anhydrous theophylline were determined.^46,47^ Furthermore, these noncovalent interactions promoted by xanthines allow the design of new heterogeneous xanthine–*X* systems (X = organic molecule, metal ion, lanthanides) which are of importance in, e.g., the synthesis of heterosynthons and cocrystals of relevance in the pharmaceutical industry.^48–53^

In this work, we present a detailed study of the intermolecular interactions underlying the dimerization of methylxanthines, accounting for the cooperative effects of both hydrogen bonding and π–stacking. A detailed description of these noncovalent interactions would allow us to better understand the behavior of these molecules in more complex arrangements, for example, in periodic crystal structures. The structural, electronic, and vibrational analyses of the monomers and dimers were performed using quantum chemical DFT methods, adding dispersive terms (DFT-D3) for a correct description of the noncovalent interactions. In addition, the chemical reactivity of the dimers was studied using global chemical descriptors. There are only a few works that address the electronic properties and chemical reactivity of some methylxanthines, limited mainly to theophylline- and theobromine-single molecules.^37,54^ Complementary analysis of noncovalent interactions (NCI) provided a deeper insight into the nature of the dimers.

## 2 Theoretical Methods

Optimization and frequency calculations were performed using Density Functional Theory (DFT), assuming a neutral charge and singlet multiplicity for all gas-phase molecules and dimers studied. The dimers were constructed in an antiparallel arrangement, based on experimental observations. The functionals and basis set used were ωB97*X*/6–311++G­(d,p), PBE/6–311++G­(d,p) and M06–2*X*/6–311++G­(d,p), as implemented in the Gaussian16 software package.^55^ The ωB97X functional has been shown to be good for the study of small noncovalent dimers.^56,57^ The M06–2X functional is suitable for unraveling noncovalent interactions in medium-sized systems.^58–60^ The M06–2X, a highly parametrized hybrid meta-GGA functional, was selected for its proficiency in describing midrange electronic correlation and capturing short-to-medium-range dispersion forces through its intrinsic functional form. To further refine the description of long-range van der Waals interactions – often underestimated by the standard functional- the M06–2X-D3 variant, incorporating Grimme’s explicit D3 dispersion correction, was also evaluated. Additional calculations were performed incorporating dispersive terms: M06–2X-D3/6–311++G­(d,p), and PBE-D3/6–311++G­(d,p). In parallel, the ωB97X range-separated hybrid (RSH) functional was employed to mitigate long-range self-interaction errors while providing a robust characterization of intermolecular potentials. Complementing these hybrid approaches, the nonempirical PBE-D3 GGA functional was used for its consistent performance in long-range regimes, where the D3 correction accurately represents the van der Waals forces governing the interlayer coupling in these stacked systems. Previous studies have shown that the dispersion-corrected PBE method can provide a fair description of systems with noncovalent interactions.^61–63^ In general, studies have shown that dispersion-corrected DFT methods significantly improve the description of molecular properties.^64,65^

All dimers were optimized under the assumption of a single multiplicity (M = 1) and a neutral charge (*Q* = 0). Vibrational frequency calculations were performed at the same level of theory to characterize the nature of the stationary points and ensure that the optimized geometries correspond to true local minima, as confirmed by the absence of imaginary frequencies.

The reactivity of methylxanthines and their dimers was studied using global molecular descriptors (chemical potential *μ*, global hardness *η*, and electrophilicity index ω), calculated from the energies of the frontier molecular orbitals HOMO and LUMO, as described by Koopmans’ theorem.^66^

1 $$\mu ={\left(\frac{\partial E}{\partial N}\right)}_{\nu \left(r\right)}=-\left(\frac{I+A}{2}\right)$$

2 $$\eta ={\left(\frac{\partial^{2}E}{\partial N^{2}}\right)}_{\nu \left(r\right)}=\frac{I-A}{2}$$

3 $$\omega =\frac{\mu^{2}}{2\eta }$$

Where *E* is the total energy, *N* is the number of electrons, v­(*r*) is the external potential of the system, *I* is the ionization potential, and *A* is the electron affinity.^67–69^ In addition, the cohesive energy (*E*_*coh*_) was calculated for all the studied systems, using the (eq 4):

4 $$E_{coh}=\frac{E_{xan/dimer}-\left(aE_{C}+bE_{H}+cE_{N}+dE_{O}\right)}{a+b+c+d}$$

Where *E*_*xan*/*dimer*_ is the total energy of the methylxanthine molecule or dimer, while *E*_*C*_, *E*_*H*_, *E*_*N*_ and *E*_*O*_ stand for the energy of the isolated carbon, hydrogen, nitrogen, and oxygen atoms, respectively. The coefficients *a*, *b*, *c*, and *d* correspond to the number of carbon, hydrogen, nitrogen, and oxygen atoms, respectively, present in the molecule. Consequently, (eq 4) yields the cohesive energy normalized per atom (*E*_*coh*_)/atom, rather than the total cohesive energy of the assembly. This parameter, expressed in eV/atom, evaluates the structural stability of the system and is defined as the average energy required to separate the molecule into its individual constituent atoms.^70–72^ Therefore, a more stable system is characterized by lower (more negative) *E*_*coh*_ values. On the other hand, the interaction energy (*E*_*int*_), (eq 5) was calculated to quantify the interaction energy between pairs of methylxanthines within the dimers.

5 $$E_{int}=E_{dimer}-2E_{xan}$$

The term 2*E*_*xan*_ arises because we are dealing with homodimers. The magnitude of the electronic interaction energy (*E*_*int*_) can be used to evaluate the strength of the assembly, where values where *E*_*int*_ > −0.5 (∼−11.5 kcal/mol), are typically associated with weaker dispersion-driven regimes, whereas values where *E*_*int*_ < −0.5 eV indicate the establishment of strong noncovalent interactions (such as cooperative hydrogen bonding and π–π stacking).^73–75^ Therefore, a more negative *E*_*int*_ value indicates a stronger electronic interaction between the methylxanthine monomers. In addition, the *Multiwfn* program was used to analyze the noncovalent interactions in the dimers, and VMD was used to plot the isosurface.^76,77^

Intermolecular interaction energies were decomposed using the Symmetry-Adapted Perturbation Theory (SAPT) at the SAPT0 level of theory.^78,79^ All calculations were performed using the Psi4 software package.^80^ The jun-cc-pVDZ basis set was employed,^81^ as it is specifically designed to provide a balanced description of noncovalent interactions, while SAPT provides an inherently BSSE-free decomposition of the interaction energy. To improve the computational efficiency of all the dimers studied here, the density fitting (DF) approximation was used to evaluate two-electron integrals, and the frozen-core approximation was applied throughout. The geometries of the five dimers analyzed here were kept at the previously optimized with the M06–2X-D3, M06–2X, PBE-D3, and ωB97X density functionals to ensure they correspond to true local minima on the potential energy surface, thereby guaranteeing a consistent description of the stationary points across all levels of theory. Additionally, to evaluate the performance of density functional theory within the SAPT framework, SAPT­(DFT) calculations were performed on representative complexes (caffeine and xanthine dimers) using the ωB97X, ωB97X-D, and PBE-D3 functionals, with the corresponding data compiled in Table S4 of the Supporting Information.

## 3 Results and Discussion

### 3.1 Structural Properties

The gas-phase optimized methylxanthine molecules did not show significant differences in bond lengths across the functional used or the dispersion corrections considered, as shown in Table S1. The bond lengths and angles obtained agree with those reported theoretically and experimentally (see Table S2).^82–85^ For the structural analysis of dimers, we focus on the distances that characterize noncovalent interactions, i.e., hydrogen bond lengths and angles, and interplanar distances and angles for π–π stacking. The structural parameters obtained with the dispersion-corrected functionals ωB97X, M06–2X, PBE-D3, and M06–2X-D3 are summarized in Table 1. Hydrogen bonds (H-bond) are in a range of 2.39–2.73 Å, 2.40–2.72 Å, and 2.50–2.70 Å obtained with ωB97*X*/6–311++G­(d,p), PBE-D3/6–311++G­(d,p) and M06–2X-D3/6–311++G­(d,p), respectively, values that agree with what is expected for C–H···O and C–H···N hydrogen bonds. H-bond distances were measured between the hydrogen atom (of a methyl group) and the acceptor atom (O or N) (see Figure S2). H-bonds formed between methyl protons and imidazole nitrogen atoms are weaker compared to those formed between methyl protons and the carbonyl oxygen atoms, since the latter have shorter lengths and larger directional angles. An 180° angle between donor–hydrogen–acceptor favors and strengthens H-bonds. The obtained average H-bond lengths are 2.43 and 2.69 Å for C=O···HC and CN···HC, respectively, with ωB97X and PBE-D3, and 2.52, and 2.68 Å for C=O···HC and CN···HC, respectively, with M06–2X, and M06–2X-D3. This suggests that both D3-functionals similarly describe the H-bonds in methylxanthine dimers. However, this is not the case with π–π stacking. The interaction distance of the π–π stacking is the distance between the centroids of each purine moiety (fused bicyclic pyrimidine-imidazole), and the planes corresponding to each purine moiety were also calculated to measure the π–π interaction angle (see Figure 2 and Figure S3). In all cases, an interaction angle of 0° is observed, indicating that both methylxanthine molecules within the dimer are arranged in a completely parallel manner. Furthermore, the centroids do not overlap, indicating a *parallel-displaced π*–π stacking. The average interplanar π–π distances are 3.53, 3.61, and 3.25 Å obtained with ωB97X, PBE-D3 and M06–2X-D3, respectively. The structural parameters predicted by M06–2X for both H-bonds and π–π stacking are very close to those obtained with M06–2X-D3 (see Table 1), indicating no significant difference between the two methods. Experimentally, π–π interplanar distances range from 3.269–3.473 Å, 3.233–3.286 Å, and 3.260–3.439 Å for caffeine, theobromine and theophylline, respectively.^32,45,86^ Therefore, the M06–2X and M06–2X-D3 functionals show better agreement with experimental values. However, it should be noted that these correspond to crystallographic data (solid state), where a complex network of noncovalent interactions beyond just a dimer, significantly modulates the distances associated with H-bonds and π–π stacking.

**Table 1:** Interaction Lengths and Angles in Hydrogen Bonds (H-Bonds) and *π*–*π* Stacking Observed in Methylxanthine Dimers Optimized with PBE-D3/6–311++G­(d,p), M06–2X-D3/6–311++G­(d,p), *ω*B97*X*/6–311++G­(d,p), and M06–2*X*/6–311++G­(d,p)^a^

Structural Properties						
PBE-D3	H-Bond and π–π Stacking Lengths and Angles in Dimers					
Dimer	d (C=O···H)	Angle	d (N···H)	Angle	d (π–π Stacking)	Angle
Dimer 1	2.442	140.1	2.681	131.4	3.694	0.02
Dimer 2	2.411	134.8	2.672	129.8	3.619	0
Dimer 3	2.407	139.1	—	—	3.673	0
Dimer 4	2.467	142.1	2.715	128.5	3.554	0
Dimer 5	—	—	—	—	3.528	0
**M06–2X-D3**						
Dimer 1	2.508	124.1	2.696	117.6	3.233	0.01
Dimer 2	2.503	114.9	2.66	119.2	3.223	0
Dimer 3	2.541	117.6	—	—	3.266	0
Dimer 4	2.535	123.3	2.683	119.7	3.238	0
Dimer 5	—	—	—	—	3.283	0
**ωB97X**						
D1	2.442	137.6	2.686	128.4	3.591	0
D2	2.405	132.9	2.680	127.2	3.533	0
D3	2.397	137.5	—	—	3.617	0
D4	2.483	139.6	2.723	126.0	3.457	0
D5	—	—	—	—	3.450	0.01
**M06–2X**						
Dimer 1	2.508	124.0	2.696	117.3	3.230	0
Dimer 2	2.502	114.9	2.658	119.1	3.219	0
Dimer 3	2.539	117.5	—	—	3.263	0
Dimer 4	2.534	123.28	2.681	119.69	3.235	0
Dimer 5	—	—	—	—	3.279	0

a Dimer 1, 2, 3, 4, and 5 correspond to caffeine–caffeine, paraxanthine–paraxanthine, theobromine-theobromine, theophylline–theophylline, and xanthine–xanthine dimers, respectively.

**Figure 2:**
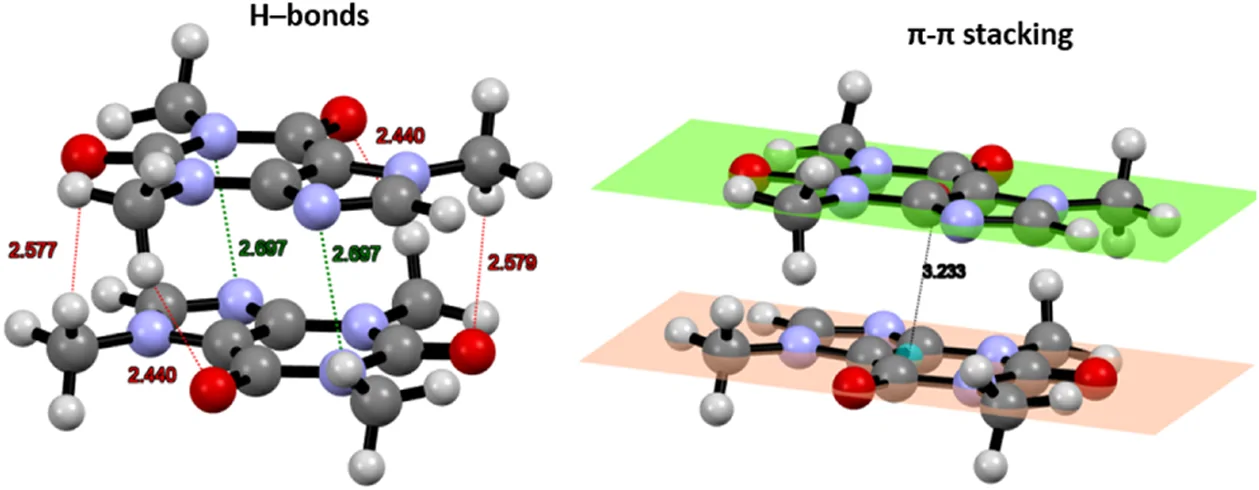
(Left) M06–2X-D3/6–311++G­(d,p) optimized caffeine dimer, showing the hydrogen bond (H-bond) interactions between monomers, distinguished by red- and green-dotted lines for d­(C=O···H) and d­(N···H) interactions, respectively. (Right) M06–2X-D3/6–311++G­(d,p) optimized caffeine dimer, showing the planes and centroids of each of the constituent planar molecules, distinguished by different colors. Interplanar distance of 3.233 Å. The number associated with the interaction is in Å units.

### 3.2 Vibrational Properties

Figures 3, S4 and S5 show the calculated IR spectrum of the methylxanthines obtained using M06–2X-D3/6–311++G­(d,p), PBE-D3/6–311++G­(d,p), and ωB97*X*/6–311++G­(d,p), respectively. The regions where the peaks corresponding to C=O, C–H, and N–H stretching appear are highlighted. The absence of peaks associated with C–H stretching for xanthine, as well as the absence of peaks corresponding to N–H stretching for caffeine, is observed. This is consistent with the fact that xanthine does not contain methyl groups, whereas caffeine has all its N-sites methylated. In all cases, the most intense band corresponds to C=O stretching (1750–1850 cm^–1^), in agreement with reported values for aromatic ketones (∼1700 cm^–1^). Likewise, the C–H (3000–3200 cm^–1^) and N–H (∼3600 cm^–1^) stretches of the calculated IR spectra are consistent with experimental values reported for other xanthines (∼3000 cm^–1^ and ∼3400 cm^–1^, respectively, see Table 2).^87–89^ Crucially, a systematic redshift in the C=O stretching frequencies is observed as a function of progressive methylation across the monomers. While the unsubstituted xanthine exhibits carbonyl bands at 1849 and 1829 cm^–1^, the addition of methyl groups induces a noticeable displacement toward lower wavenumbers, with the dimethylated isomers appearing at intermediate regions (e.g., 1818 and 1776 cm^–1^ for theophylline), and the trimethylated caffeine displaying the lowest frequencies at 1812 and 1771 cm^–1^. This trend is directly attributed to the electron-donating inductive effect (+I) of the methyl substituents, which enhances electron density delocalization onto the carbonyl groups, lowering their bond force constants. This predictable frequency modulation offers an essential theoretical framework for distinguishing these structural analogs via linear vibrational spectroscopy and advanced two-dimensional infrared (2D-IR) techniques. The IR spectra calculated using the M06–2X-D3 and ωB97X functionals are very similar to each other, whereas those obtained with PBE-D3 exhibit a slight blue shift in their peak positions (see Figure 4).

**Table 2:** Wavenumber Values of the Main Vibrational Modes Calculated for (Methyl)­xanthine Obtained from Frequency Calculations Using the DFT/M06–2X-D3/6–311++G­(d,p) Method

Calculated Vibrational Frequencies (cm^–1^) with M06–2X-D3					
Principal Vibrational Modes of Monomers					
Molecule	C–H Stretching	C=O Stretching	C–C Stretching	C–H Bending	N–H Stretching
Caffeine	3089–3093	1812	1669	1503	—
	3159–3182	1771			
Paraxanthine	3091–3094	1830	1668	1634	3664
	3162–3217	1753			
Theobromine	3089–3092	1823	1660	1200–1550	3632
	3160–3201	1812			
Theophylline	3091–3093	1818	1680	1200–1550	3681
	3161–3220	1776			
Xanthine	—	1849	1668	—	3628–3679
		1829			

**Figure 3:**
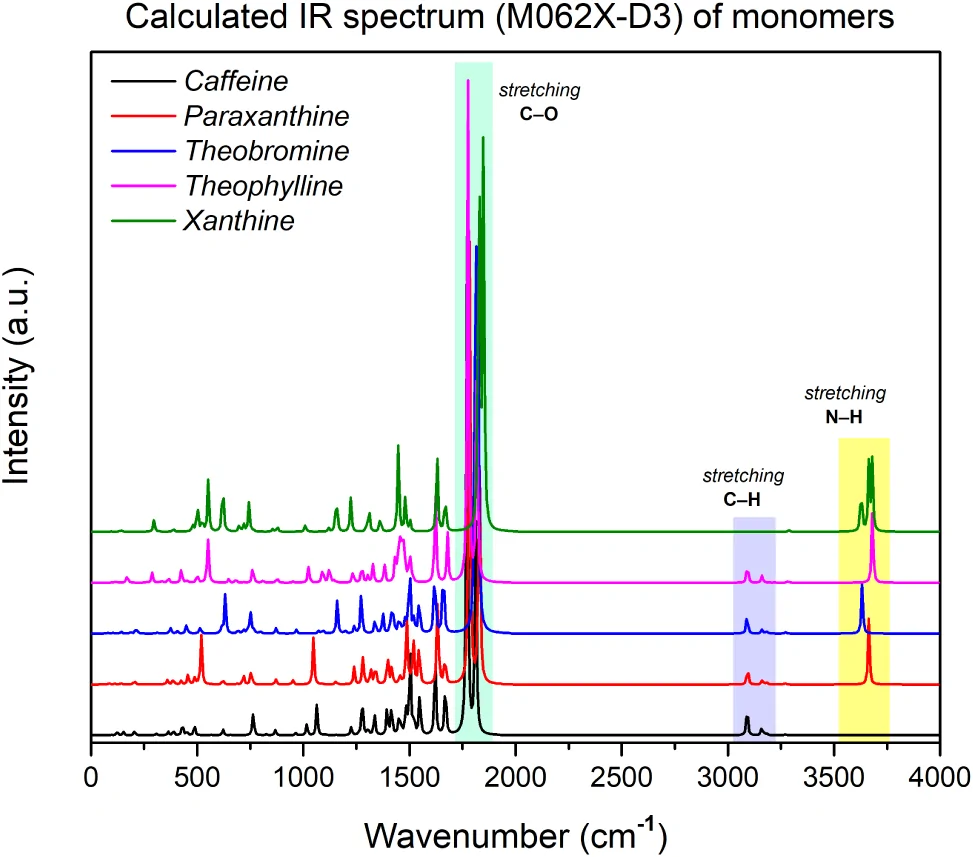
Theoretical IR spectra of caffeine (black), paraxanthine (red), theobromine (blue), theophylline (pink) and xanthine (green), from 0 cm^–1^ to 4000 cm^–1^ using DFT/M06–2X-D3/6–311++G­(d,p).

**Figure 4:**
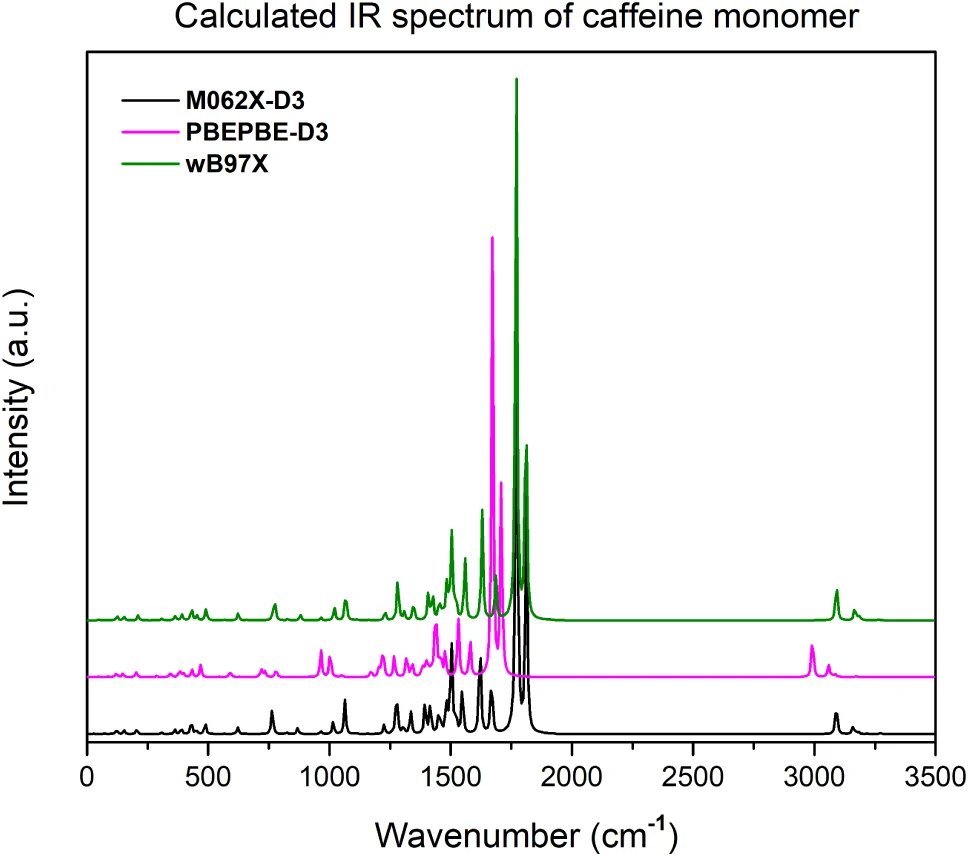
Theoretical IR spectra of caffeine from 0 cm^–1^ to 3500 cm^–1^ using DFT/M06–2X-D3/6–311++G­(d,p) (black), PBE-D3/6–311++G­(d,p) (pink) and ωB97*X*/6–311++G­(d,p) (green).

Figure 5 compares the calculated IR spectra of the caffeine monomer and dimer, showing that they are very similar. The dimer exhibits additional low-intensity bands in the low-frequency region (<1000 cm^–1^), which can be attributed to both inter- and intramolecular vibrational modes. In addition, a slight *redshift* is observed in the bands associated with the C–O and N–H stretching when going from the monomer to the dimer, caused by the hydrogen bonds present in the dimer, where these functional groups (carbonyl and amino) participate. The redshift of IR peaks from a *free* molecule (without intermolecular interactions) can be indicative of noncovalent interactions and provide a semiquantitative measure of their strength, particularly in the case of hydrogen bonds.^90,91^ The IR spectra of all the dimers studied are shown in Figure S6.

**Figure 5:**
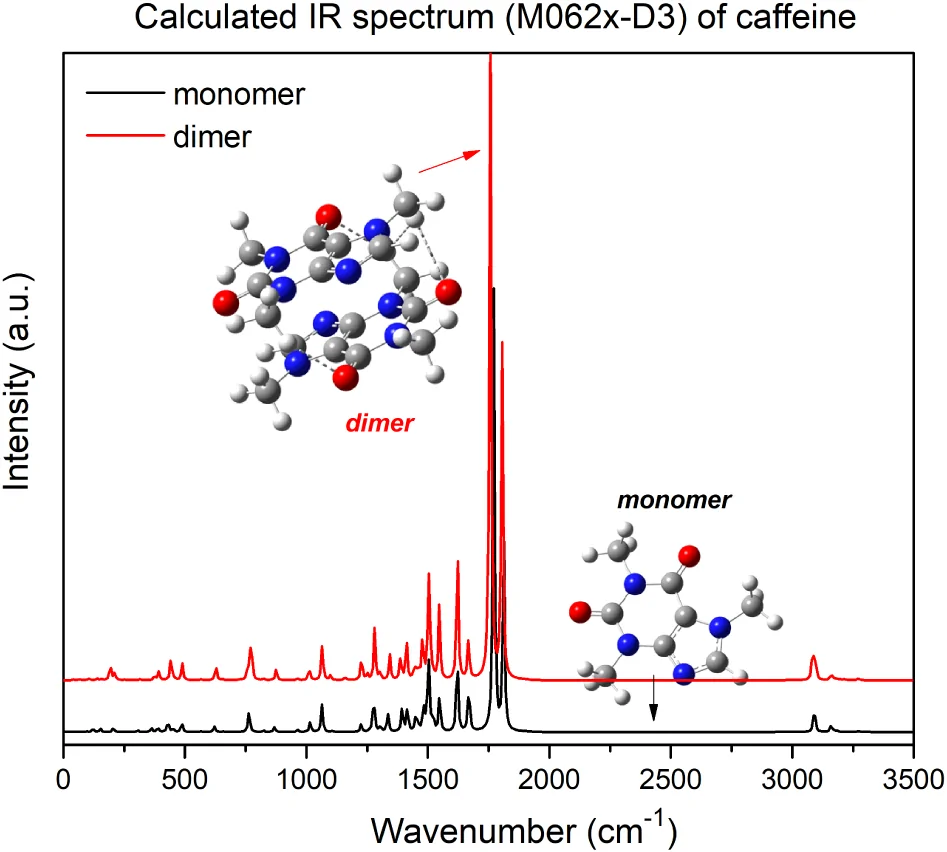
Theoretical IR spectra of caffeine monomer (black) and dimer (red) from 0 cm^–1^ to 3500 cm^–1^ using DFT/M06–2X-D3/6–311++G­(d,p) (Inside: optimized molecules of caffeine monomer and dimer).

### 3.3 Electronic Properties

#### 3.3.1 Frontier Molecular Orbitals, Interaction Energy, and Cohesive Energy

The stability and reactivity of the complexes are determined using global molecular descriptors, such as chemical potential (*μ*), global hardness (*η*), and electrophilicity index (*ω*), as well as the HOMO–LUMO energy gap (*E*_*gap*_), cohesive energy (*E*_*coh*_), and interaction energy (*E*_*int*_). Tables S3–S5 and Tables 3–4,5 summarize these quantities for monomers and dimers, respectively. Figures S7–S9 summarize the frontier molecular orbitals (FMOs) HOMO and LUMO and the molecular electrostatic potentials (MEP) plots of the monomers and dimers, with all the functionals used in this work. For monomers (Figure S7), the HOMO is mainly located on the carbonyl oxygen (*p* orbitals) and on delocalized π–orbitals of the purine fragment; while LUMO is mainly distributed on π^*^–orbitals (antibonding) of C=C and C=N bonds. Figure S8 highlights the importance of including dispersive terms particularly in the PBE functional, since PBE/6–311++G­(d,p) calculations did not yield a description of the xanthine dimer. Interestingly, in the case of dimers calculated through dispersive-functionals (Figure S9), it is observed that the HOMO and LUMO are distributed along the entire dimer, indicating an effective *orbital overlap* of the constituent monomers, thus suggesting a strong interaction. The *E*_*int*_ values confirm the existence of a strong interaction between xanthine molecules in the formation of the dimer. In all cases, the *E*_*int*_ < −0.5 eV (∼−11.5 kcal/mol), which indicates the establishment of strong noncovalent interactions between methylxanthine molecules, with the caffeine dimer having the highest *E*_*int*_ values (−0.993 eV [∼−22.9 kcal/mol] with M06–2X-D3/6–311++G­(d,p)), while the xanthine dimer features the lowest (−0.731 eV [∼−16.9 kcal/mol] with M06–2X-D3/6–311++G­(d,p)). It is worth noting that the *E*_*int*_ values obtained with PBE-D3 are lower (around 19–26%) compared to those obtained with M06–2X-D3 (cf. Tables 3 and 4), although in both cases the values correspond to strong noncovalent interactions. This differs from what has been observed in recent work, where the interaction energy obtained with PBE-D3 methods is higher compared to M06–2X-D3;^92,93^ however, this depends largely on the nature of the system being modeled. The *E*_*int*_ values obtained with ωB97X lie between those obtained with the other two functionals (Table 5). Interestingly, a trend is observed between *E*_*int*_ and the number of methyl substituents per xanthine molecule (see Figure S10), where a higher number of methyl groups is associated with a higher (more negative) electronic interaction energy. It is important to emphasize that this methylation trend applies strictly to the electronic interaction energy (*E*_*int*_) and does not reflect the thermodynamic stability trends (Δ*G*) discussed later. This electronic stabilization occurs because the additional methyl groups drive a dual synergistic effect: they dramatically increase molecular polarizability, which reinforces dispersion-driven π–π stacking, while concurrently increasing the number of auxiliary intermolecular hydrogen bonds within the dimeric architecture. Previous experimental work shows that increasing the number of methylated sites favors π–π stacking over hydrogen bonding (see Section 1).^31,32^ However, this occurs in the solid state, where methylation affects not only coplanar dimerization but the entire supramolecular network. Therefore, it appears that a greater number of methyl groups favors coplanar dimerization in an initial growth stage (through hydrogen bonding, as our results suggest), although they subsequently inhibit hydrogen bond interactions with adjacent dimers, thus favoring π–π stacking in a second growth stage of the supramolecular network.

**Table 3:** Optimized Total Energy (*E*_*T*_), Energy of the Frontier Molecular Orbitals (HOMO and LUMO), Molecular Gap Energy (*E*_*g*_), Global Molecular Descriptors (*η*, *μ*, *ω*, *ω* ^±^, *N″*), Cohesive Energy (*E*_*coh*_) and Interaction Energy (*E_int_*) for the Methylxanthine Dimers^a^

Methylxanthines					
Molecular Descriptor	Caffeine	Paraxanthine	Theobromine	Theophylline	Xanthine
*E*_*T*_ ^b^	–37.009	–34.8712	–34.8713	–34.871	–30.595
*E*_*HOMO*_	–7.5499	–7.8096	–7.6829	–7.6924	–8.2013
*E*_*LUMO*_	–0.3400	–0.4615	–0.4955	–0.4490	–0.7936
*E*_*g*_	7.2099	7.3480	7.1873	7.2433	7.4076
*I* = −*E*_*HOMO*_	7.5499	7.8096	7.6829	7.6924	8.2013
*A* = −*E*_*LUMO*_	0.3400	0.4615	0.4955	0.4490	0.7936
*η* = (*I* – *A*)/2	3.6049	3.6740	3.5936	3.6216	3.7038
*μ* = −(*I* + *A*)/2	–3.9449	–4.1356	–4.0892	–4.0707	–4.4975
*ω* = *μ* ^2^/2*η*	2.1588	2.3275	2.3265	2.2877	2.7306
*E*_*coh*_	–5.9897	–6.1605	–6.1637	–6.1595	–6.7101
*E*_*int*_	–0.9933	–0.8895	–0.9285	–0.8776	–0.7314

a Optimization was performed via DFT/M06–2X-D3/6–311++G­(d,p).

b Total energy is in units of keV; the rest of the parameters are given in eV.

**Table 4:** Optimized Total Energy (*E*_*T*_), Energy of the Frontier Molecular Orbitals (HOMO and LUMO), Molecular Gap Energy (*E*_*g*_), Global Molecular Descriptors (*η*, *μ*, *ω*, *ω* ^±^,*N″*), Cohesive Energy (*E*_*coh*_) and Interaction Energy (*E*_*int*_) for the Methylxanthine Dimers^a^

Methylxanthines					
Molecular Descriptor	Caffeine	Paraxanthine	Theobromine	Theophylline	Xanthine
*E*_*T*_ ^b^	–36.9815	–34.8455	–34.8456	–34.8454	–30.5735
*E*_*HOMO*_	–5.3904	–5.6257	–5.5512	–5.5050	–5.9810
*E*_*LUMO*_	–2.0587	–2.1281	–2.1855	–2.1596	–2.4945
*E*_*g*_	3.3317	3.4976	3.3265	3.3453	3.4864
*I* = −*E*_*HOMO*_	5.3904	5.6257	5.5512	5.5050	5.9810
*A* = −*E*_*LUMO*_	2.0587	2.1281	2.155	2.1596	2.4945
*η* = (*I* – *A*)/2	1.6658	1.7488	1.6632	1.6726	1.7432
*μ* = −(*I* + *A*)/2	–3.7246	–3.8769	–3.8488	–3.8323	–4.2377
*ω* = *μ* ^2^/2*η*	4.1638	4.2973	4.4530	4.3902	5.1509
*E*_*coh*_	–5.9825	–6.1353	–6.1388	–6.1333	–6.6262
*E*_*int*_	–0.7789	–0.6966	–0.7442	–0.6533	–0.5469

a Optimization was performed via DFT/PBE+D3/6–311++G­(d,p).

b Total energy is in units of keV; the rest of the parameters are given in eV.

**Table 5:** Optimized Total Energy (*E*_*T*_), Energy of the Frontier Molecular Orbitals (HOMO and LUMO), Molecular Gap Energy (*E*_*g*_), Global Molecular Descriptors (*η*, *μ*, *ω*, *ω* ^±^,*N″*), Cohesive Energy (*E*_*coh*_) and Interaction Energy (*E*_*int*_) for the Methylxanthine Dimers^a^

Methylxanthines					
Molecular Descriptor	Caffeine	Paraxanthine	Theobromine	Theophylline	Xanthine
*E*_*T*_ ^b^	–37.014	–34.875	–34.875	–34.875	–30.598
*E*_*HOMO*_	–8.5239	–8.7972	–8.6596	–8.6860	–9.2072
*E*_*LUMO*_	0.9487	0.8853	0.8176	0.8296	0.4993
*E*_*g*_	9.4726	9.6826	9.4772	9.5156	9.7065
*I* = −*E*_*HOMO*_	8.5239	8.7972	8.6596	8.6860	9.2072
*A* = −*E*_*LUMO*_	–0.9487	–0.8853	–0.8176	–0.8296	–0.4993
*η* = (*I* – *A*)/2	4.7363	4.8413	4.7386	4.7578	4.8532
*μ* = −(*I* + *A*)/2	–3.7876	–3.9559	–3.9210	–3.9282	–4.3539
*ω* = *μ* ^2^/2*η*	1.5144	1.6162	1.6222	1.6216	1.9529
*E*_*coh*_	–5.8952	–6.0647	–6.0678	–6.0636	–6.6096
*E*_*int*_	–0.8885	–0.8255	–0.8626	–0.7863	–0.6942

a Optimization was performed via DFT/ωB97*X*/6–311++G­(d,p).

b Total energy is in units of keV; the rest of the parameters are given in eV.

#### 3.3.2 Symmetry-Adapted Perturbation Theory Analysis

A comparative analysis across the different density functionals reveals a consistent trend in the stability of the studied systems, with the caffeine dimer emerging as the most stable and the xanthine dimer as the least stable in all cases (Figure 6 and Table S3, see Supporting Information). Despite this qualitative agreement, quantitative differences arise in the description of specific interaction components. Specifically, the M06–2X and M06–2X-D3 functionals predict significantly higher exchange and dispersion energies that PBE-D3 and ωB97X, suggesting a more compact intermolecular contact in the meta-GGA hybrid models. Nevertheless, the convergence of the total interaction energy across all levels of theory (≈ −17 to −18 kcal/mol for caffeine dimer) provides strong computational evidence that the methylation of the purine core is the primary factor enhancing the π–π stacking strength and overall thermodynamic stability of the caffeine dimer relative to xanthine.

**Figure 6:**
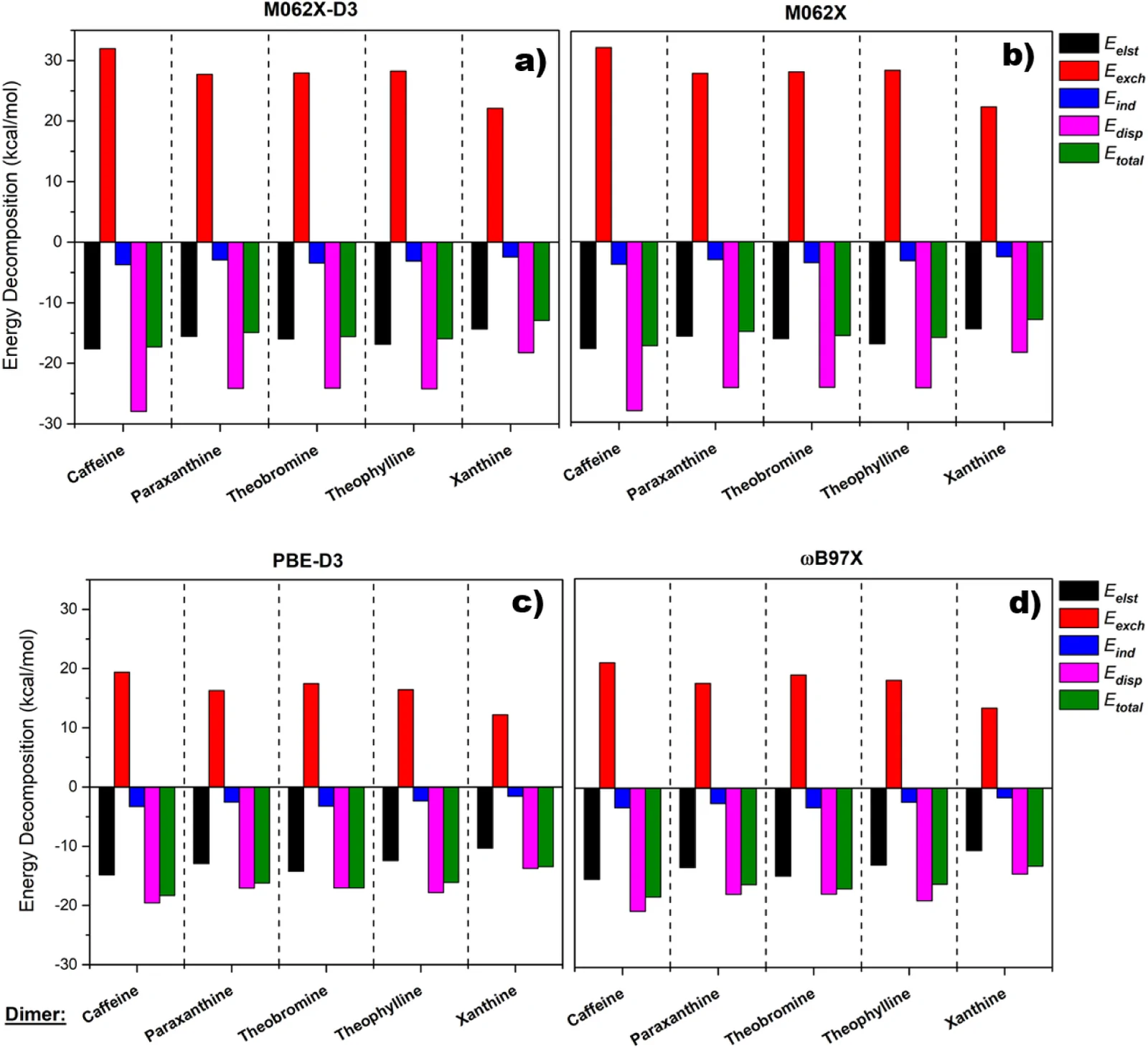
Energy decomposition analysis of the methylxanthine dimers calculated at the standard SAPT0/jun-cc-pVDZ level. The calculations were performed on the molecular geometries previously optimized with the following density functionals: (a) M06–2X-D3, (b) M06–2X, (c) PBE-D3 and (d) ωB97X. *E*_*elst*_ = electrostatic energy; *E*_*exch*_ = exchange energy; *E*_*ind*_ = induction energy; *E*_*disp*_ = dispersion energy; *E*_*total*_ = total SAPT0 interaction energy.

The induction component (*E*_*ind*_) across all studied dimers remains consistently low, ranging from −1.5 to −3.7 kcal/mol, representing the smallest contribution to the total attractive force (less than 10% in most cases). This finding is of particular interest as it confirms the strictly noncovalent nature of the interaction. The negligible magnitude of *E*_*ind*_ suggests that there is no significant charge transfer or substantial polarization of the electron clouds between the monomeric units. Consequently, the stability of these systems is governed almost exclusively by a synergy between electrostatic forces and London dispersion forces, rather than by orbital-driven interactions or Lewis acid–base behavior. This explains why traditional frontier molecular orbital (FMO) analysis often fails to rationalize the binding in methylxanthine dimers, as their association is dictated by long-range physical forces rather than chemical reactivity. The comparative analysis across the four density functionals under the SAPT0 level of theory reveals a remarkable physicochemical consistency in describing the interaction profile of methylxanthines. Regardless of the functional employed, London dispersion (*E*_*disp*_) emerges as the dominant attractive force, consistently accounting for 50% of the total attraction throughout the series. This uniform trend underscores that while electrostatics may serve as a structural director, the thermodynamic stability of these dimers is primarily governed by π–π stacking. Furthermore, the invariance in the stability order (D1 > D4 > D3 > D2 > D5) is invariant across all methods, validating the robustness of the SAPT0 framework in rationalizing how progressive methylation of the purine core enhances van der Waals forces, thereby optimizing the overall interaction energy.

To validate the methodological framework, a benchmark comparison between SAPT0 and SAPT­(DFT) is presented in the Supporting Information (Table S4). This analysis, focused on the caffeine and xanthine dimers, supports the reliability of the energy decomposition for the studied methylxanthines.

#### 3.3.3 Global Molecular Descriptors

Global hardness (*η*) and chemical potential (*μ*) are involved within the ω descriptor, the electrophilicity index, which measures a molecule’s ability to accept electrons in an electron-rich environment.^68,69^ Therefore, a good electrophile (high ω value) will be characterized by high *μ* values and low *η* values. The global molecular descriptors and *E*_*coh*_ values of the monomers are very close to those of the dimers, indicating that dimerization does not affect the reactivity or stability of the constituent (methyl)­xanthines (cf. Tables 3 and S5). Furthermore, methylation subtly decreases the electrophilic character of the dimers, with the pristine xanthine-dimer being the most electrophilic system (*ω*) = 2.73 eV, M06–2X-D3. It is observed that the functional has a significant impact on the magnitude of the global molecular descriptors, although it does not affect the trends observed.

### 3.4 Noncovalent Interactions (NCI)

NCI analysis is useful for studying inter- and intramolecular interactions by identifying high-density regions. In the graphs of Figures 7 and S11, the *X*-axis and *Y*-axis correspond to *sign*(*λ*_2_)*ρ* and RDG, respectively. In the NCI theory, reduced density gradient (RDG) graphs are visual representations to show the relative strength of different types of interactions, while *sign*(*λ*_2_)*ρ* is related to the sign (and type) of the interactions: when the sign of the eigenvalue is negative, *λ*_2_ < 0, it indicates mainly hydrogen bonds, if *λ*_2_ ≈ 0 it is related to van der Waals interactions, and *λ*_2_ > 0 corresponds to steric effect interactions. The RDG function shown in eq 6 is defined as a dimensionless form of the electron density gradient norm function.

6 $$\mathrm{RDG}\left(r\right)=\frac{1}{2{\left(3\pi^{2}\right)}^{1/3}}\frac{\left|\nabla \rho \left(r\right)\right|}{\rho {\left(r\right)}^{4/3}}$$

**Figure 7:**
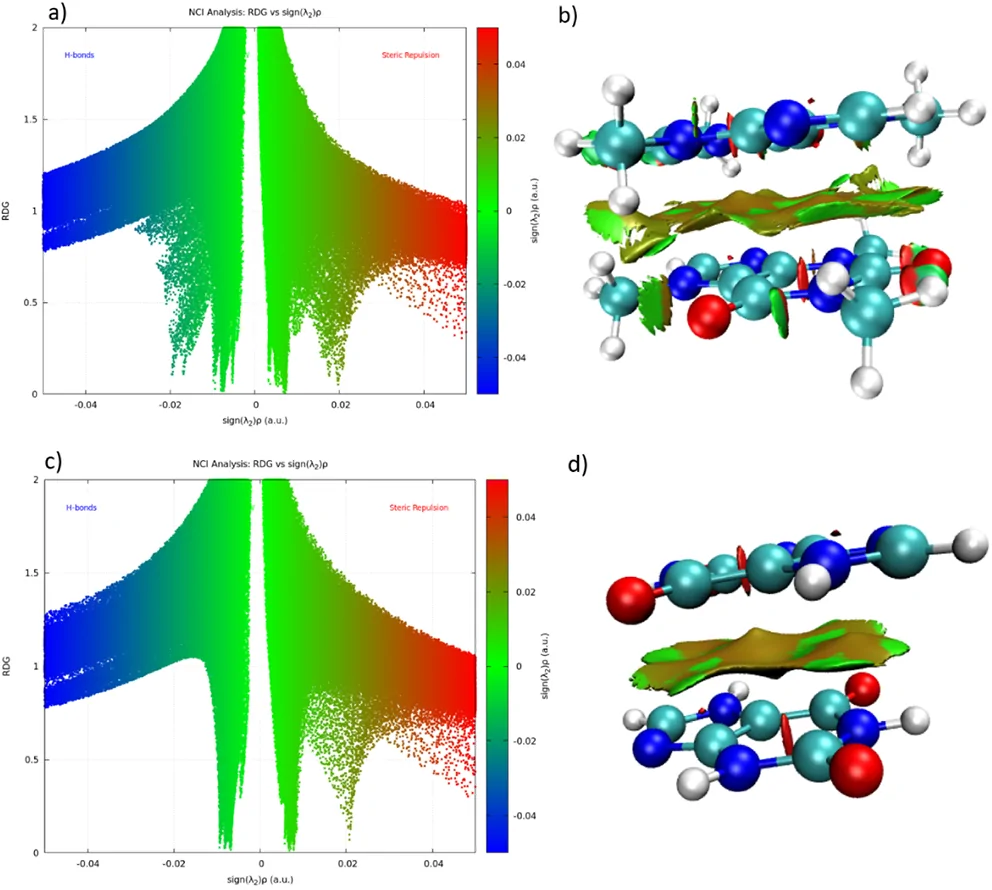
RDG vs *sign*(*λ*_2_)*ρ* plots and intermolecular interactions (the RDG isosurface is 0.5) of (a, b) caffeine and (c, d) xanthine dimers. Color code for the isosurface: H-bonds (blue), vdW (green), steric (red).

The RDG vs *sign*(*λ*_2_)*ρ* graphs show that in methylxanthine dimers there are contributions from both hydrogen bonds and steric effects; however, the region of greatest density (main interaction) corresponds to van der Waals (vdW) type interactions. When analyzing the graph of the pristine xanthine dimer (without methyls, Figure 7c), we observe the absence of RDG density in the hydrogen bonding region, indicating that it is indeed these methyl groups that promote the H-bonds. Figure 7 also shows the isosurface plots of the caffeine- and xanthine-dimers, respectively, which are located between both methyl­(xanthine) molecules, suggesting a vdW interaction by π–π stacking. In addition, steric effects (isosurface in red) are observed within the aromatic purine moiety.

### 3.5 Thermochemistry

Gibbs free energies of dimerization (Δ*G*_*Dimer*_) were calculated to provide both qualitative and quantitative insights into the self-association behavior of the investigated methylxanthines. Table 6 summarizes these values, determined according to eq 7:

7 $$\Delta G_{\mathrm{Dimer}}=G_{\mathrm{dimer}}-2G_{\mathrm{monomer}}$$

**Table 6:** Calculated Gibbs Free Energies of Dimerization (ΔG_Dimer_, in kcal/mol) for Caffeine and Related Methylxanthines at 298.15 K and 1 atm

Functional	Caffeine	Paraxanthine	Theobromine	Theophylline	Xanthine
M06–2X-D3	–1.91	–4.16	–4.88	–6.45	–2.90
PBE-D3	–1.91	–2.84	–1.54	–1.36	–0.85
M06–2X	–3.46	–2.64	–3.36	–3.44	–2.00
ωB97X	+1.09	+2.07	–0.36	+1.71	+2.43

The thermochemical contributions were evaluated under ambient conditions (298.15 K and 1 atm) utilizing the standard rigid-rotor harmonic-oscillator (RRHO) approximation. Our results highlight the critical role of dispersion corrections in stabilizing the stacked architectures.^65^ The comparative thermodynamic data across different functionals, including the effect of D3 corrections, reflects the high sensitivity of these weakly bound complexes to the chosen density functional.

Upon closer inspection of the Δ*G*_*Dimer*_ values, the predicted stability order heavily depends on the density functional utilized. Specifically, while the uncorrected M06–2X functional positions the caffeine dimer as the most stable (−3.46 kcal/mol), the dispersion-corrected M06–2X-D3 predicts the theophylline dimer to be significantly more stable (−6.45 kcal/mol). Conversely, under PBE-D3, caffeine and paraxanthine share the highest stability (−1.91 kcal/mol), whereas ωB97X yields positive Δ*G* values for most species except theobromine. The positive Δ*G* values obtained with the ωB97X functional suggest an underestimation of the long-range dispersion forces in these stacked purine systems. Furthermore, this behavior is amplified by the standard RRHO approximation, which is known to overestimate the entropic penalty arising from low-frequency intermolecular vibrational modes in gas-phase dimers. In contrast, the M06–2X-D3 and PBE-D3 results show better agreement with the trends observed in experimental self-association studies.^94^ Notably, under the M06–2X-D3 functional, the theophylline dimer exhibits the highest thermodynamic stability (Δ*G* = −6.45 kcal/mol), surpassing caffeine. In the absence of solvent, the bulkier methyl groups of caffeine introduce local steric constraints and a more severe vibrational entropic penalty compared to less-substituted xanthines like theophylline, which benefits from a less hindered interplanar approach. Furthermore, it is worth noting that while experimental self-association studies in aqueous solution typically show high stacking propensities for caffeine due to the hydrophobic effect, our gas-phase models isolate the intrinsic molecular forces. This highlights the delicate interplay between electronic stabilization and entropic factors in isolated supramolecular assemblies. These findings underscore that while electrostatics direct the orientation, the thermodynamics spontaneity is largely driven by dispersion-stabilized stacking. Although the calculated interaction energies follow different trends across the functionals, a definitive benchmarking against experimental values remains elusive due to the lack of gas-phase dimerization data. Nevertheless, the qualitative insights provided by these methods highlight the critical role of dispersion corrections in describing these xanthine-based complexes.

## 4 Conclusions

This study provides a comprehensive characterization of methylxanthine dimers using DFT-D3 and SAPT0 analysis. Our results validate that dimerization is a spontaneous process, as demonstrated by the calculated Gibbs free energies. A key finding is that London dispersion is essential, acting as the primary force stabilizing the π–π stacking; without it, the overall stability of the dimers is significantly underestimated. The SAPT0 decomposition identifies a synergistic interplay between electrostatic-driven hydrogen bonding and dispersion-driven stacking. The wide HOMO–LUMO gaps (7.2–9.4 eV) reflect high electronic localization, correlating with a minimal induction contribution (*E*_*int*_) < 9.5%) and negligible charge transfer. Notably, the thermodynamic stability trends (Δ*G*) were found to be highly dependent on the choice of density functional, rather than following a strict monotonic order based on the degree of methylation. Furthermore, the color-coded NCI-RDG analysis provides an unambiguous mapping of the competing attractive and steric forces. While dimerization preserves the global reactivity descriptors of the monomers, the interaction energies confirm a stable self-assembly regime governed by strong noncovalent interactions. These fundamental insights provide a valuable molecular framework for understanding the self-assembly of methylxanthine-based systems. While future studies incorporating solvent effects and crystal packing are required to fully model condensed phases, this work establishes a rigorous gas-phase baseline that contributes to the broader understanding of supramolecular materials and cocrystals.

### 4.1 Software and Hardware Details

All calculations were performed with the software Gaussian 16,^55^ Revision B.01, by means of two processors (Intel Xeon E5-2680v3 and 30 MB cache, 2.50 GHz, and 24 cores with a total RAM of 512 GB).

## Supplementary Material



## Notes

Optimized bond lengths, atomic numbering schemes, and structures of caffeine monomers and dimers; theoretical IR spectra, molecular electrostatic potential (MEP) isosurfaces, and HOMO/LUMO spatial distributions for methylxanthines; SAPT analysis data, optimized total energies, global molecular descriptors, and reduced density gradient (RDG) analysis plots (PDF)
