# Supplementary material for: Theoretical Analysis of Electronic, Vibrational Properties, and Noncovalent Interactions in Methylxanthines: Monomers to Antiparallel Dimers
Source: ACS Omega. 2026 Aug 10;11(33):49268–79. doi: 10.1021/acsomega.6c02053 (PMC13520041; doi:10.1021/acsomega.6c02053)
Supplement: Supplementary file 1 [file ao6c02053_si_001.pdf]

Theoretical Analysis of Electronic, Vibrational Properties and Non-Covalent Interactions in Methylxanthines: Monomers to Antiparallel Dimers

Raúl Mendoza-Báez[1], Dolores Garcia-Toral [2]* Alexandra Deriabina[3], María Patricia Sánchez Gutiérrez[3], Víctor M. Vázquez-Báez[4], Akari Narayama Sosa Camposeco[5], Alexandra Bustamante Camacho [5], Gregorio Hernández Cocoletzi[5] and Juan Francisco Rivas-Silva[5].

[1] Departamento de Química, Centro de Investigación y de Estudios Avanzados del IPN (Cinvestav), Avenida IPN 2508, Col. San Pedro Zacatenco, Ciudad de México 07360, México.

raul.mendoza@cinvestav.mx

[2] Facultad de Ingeniería Química, Benemérita Universidad Autónoma de Puebla, Av. San Claudio y 18 Sur S/N, San Manuel, Puebla 72570, México.

dolores.garcia@correo.buap.mx

[3] Facultad *de Ciencias Físico Matemáticas, Benemérita Universidad Autónoma de Puebla, Puebla 72570, México*

aderiabina@fcfm.buap.mx, patricia.sg@fcfm.buap.mx

[4] Facultad de Ingeniería, Benemérita Universidad Autónoma de Puebla, Puebla 72570, México.

manuel.vazquez@correo.buap.mx

[5] *Instituto de Física "Ing. Luis Rivera Terrazas", Benemérita Universidad Autónoma de Puebla, Av. San Claudio y Blvd. 18 sur, Col. San Manuel, Ciudad Universitaria, Puebla, Pue. 72570, México.*

asosa@ifuap.buap.mx, alexabc@ifuap.buap.mx, cocoletz@ifuap.buap.mx, rivas@ifuap.buap.mx

Corresponding author: D. Garcia-Toral (dolores.garcia@correo.buap.mx)*

Supplementary Information

Table S1. Selected bond lengths (Å) for the optimized caffeine molecule (as example) with ωB97X, PBE, PBE-D3, M06-2X, and M06-2X-D3. The basis set used was 6-311++g(d,p) for all cases. Atom numbering associated with Figure S1.

Selected bond lengths in the optimized caffeine molecule

Bond	**ωB97X**	**PBE**	**PBE-D3**	**M06-2X**	**M06-2X-D3**
N_1–C_{11}	1.462	1.466	1.466	1.463	1.463

N_3–C_{16}	1.457	1.463	1.463	1.458	1.459
N_7–C_{21}	1.453	1.459	1.459	1.455	1.455
N_{10}–C_8	1.323	1.336	1.337	1.325	1.325
C_2–O_{15}	1.214	1.228	1.228	1.212	1.212
C_6–O_{20}	1.218	1.234	1.234	1.216	1.216
C_4–C_5	1.369	1.391	1.391	1.373	1.373

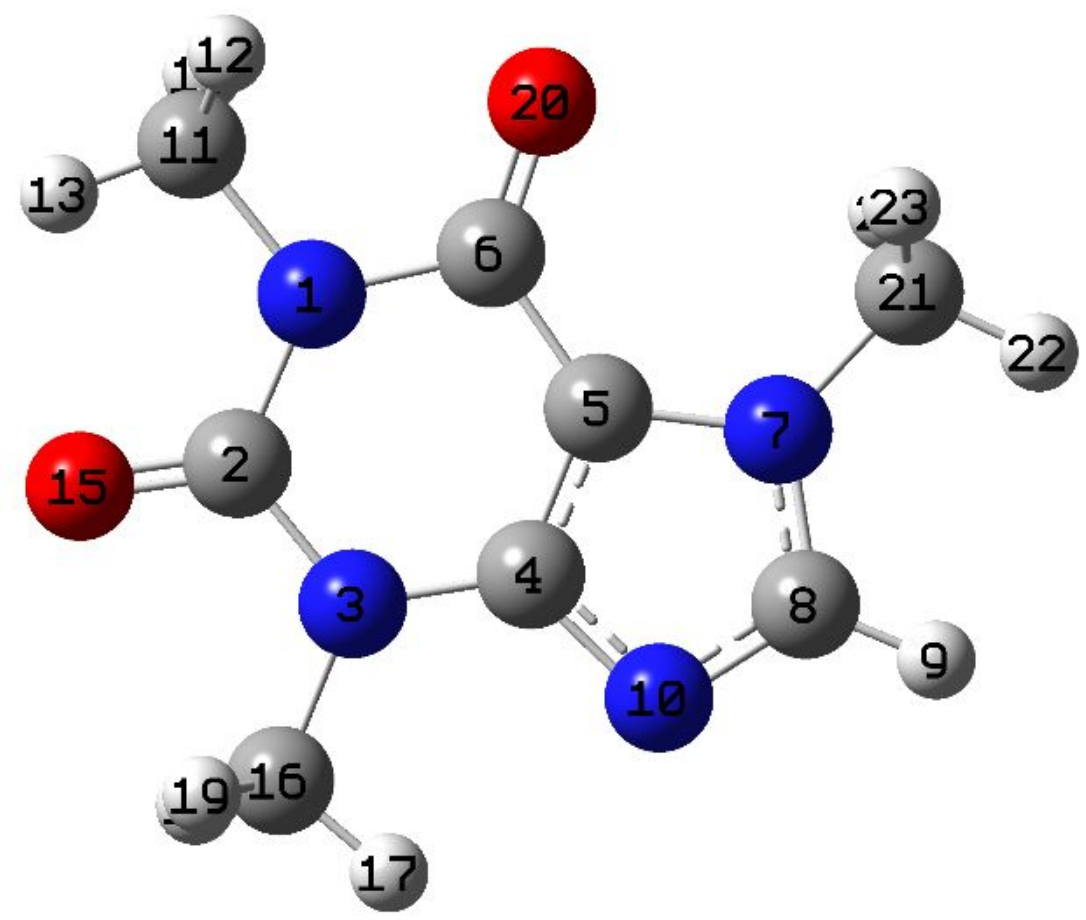

Figure S1. Atomic numbering of the caffeine molecule optimized with ωB97X/6-311++g(d,p). The numbering is the same for each functional used.

Table S2. Selected bond lengths (Å) for the optimized caffeine molecule (as an example) with ωB97X, PBE-D3, and M06-2X-D3 compared with other theoretical (B3LYP-D3, Hartree-Fock) and experimental values. The basis set used in this work was 6-311++g(d,p). Atom numbering associated with Figure S1.

Comparison of bond lengths in the optimized and experimental caffeine molecule

Bond	**ωB97X**	**PBE-D3**	**M06-2X-D3**	**B3LYP-D3[a]**	**HF[b]**	**Exp[c]**	**Exp[d]**
N_1–C_{11}	1.462	1.466	1.464	1.464	1.460	1.469	1.48
N_3–C_{16}	1.457	1.463	1.459	1.458	1.460	1.464	1.50
N_7–C_{21}	1.453	1.459	1.455	1.456	1.460	1.459	1.47
C_8–H_9	1.082	1.088	1.080	1.080	—	1.085	1.15

[a] Ref[76], [b] Ref[77], [c] Ref[78], d Ref[79].

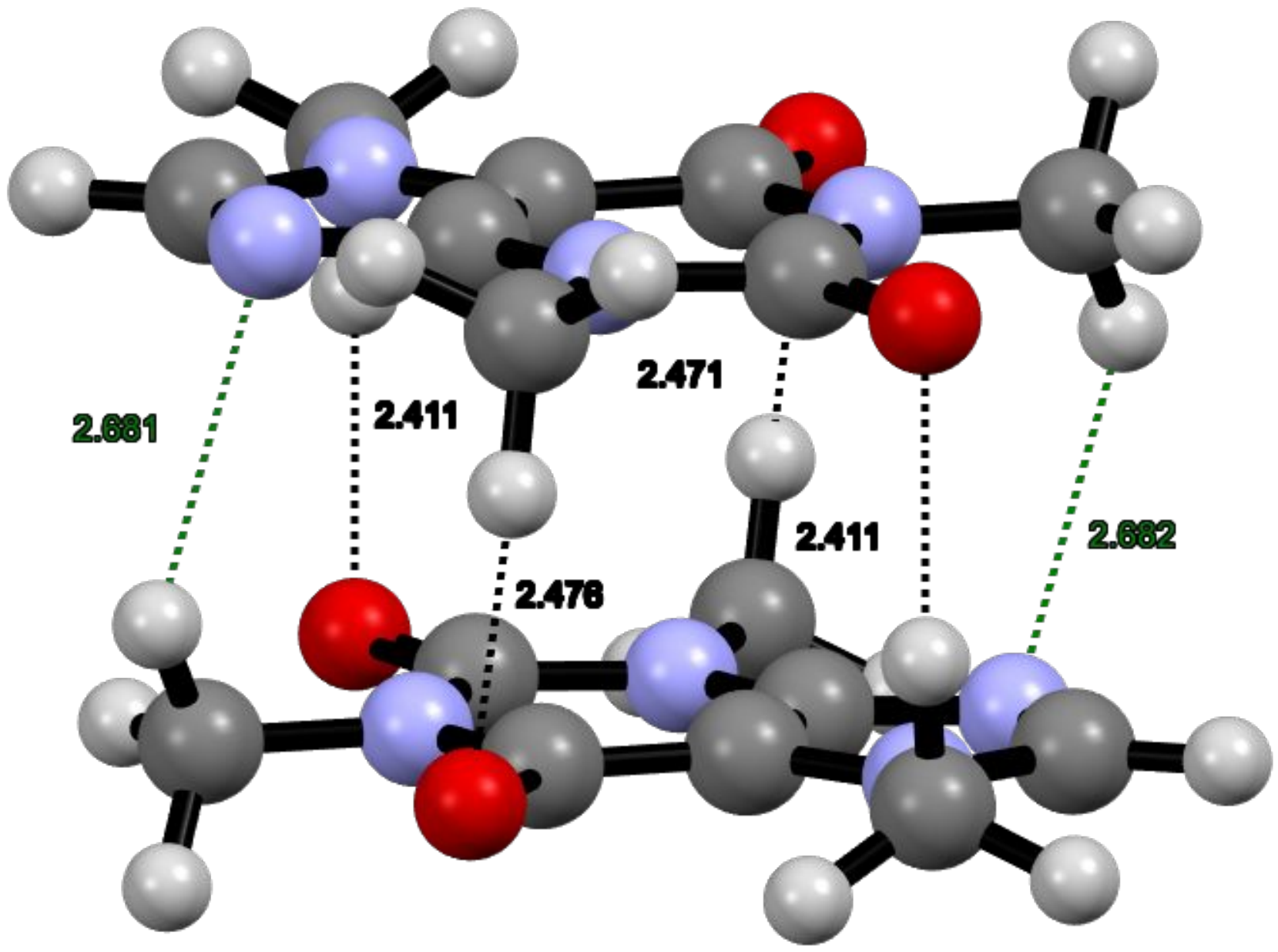

Figure S2. PBE-D3/6-311++g(d,p) optimized caffeine dimer, showing the hydrogen bond (H-bond) interactions between monomers, distinguished by green- and black-dotted lines for d(C=O•••H) and d(N•••H) interactions, respectively. The number associated with the interaction is in Å units.

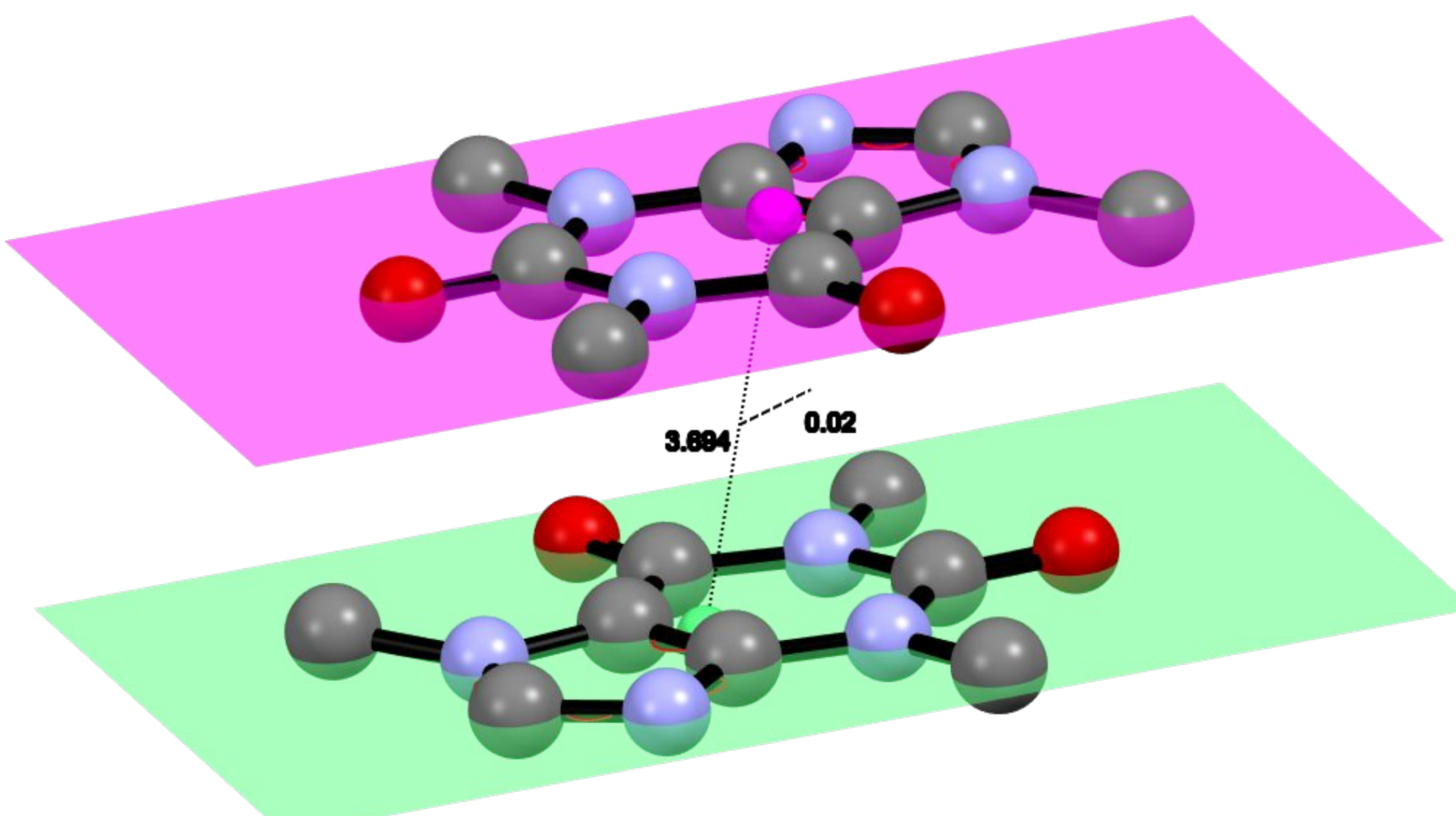

Figure S3. PBE-D3/6-311++g(d,p) optimized caffeine dimer, showing the planes and centroids of each of the constituent planar molecules, distinguished by green and pink colors. Interplanar distance of 3.694 Å and angle of 0.02°.

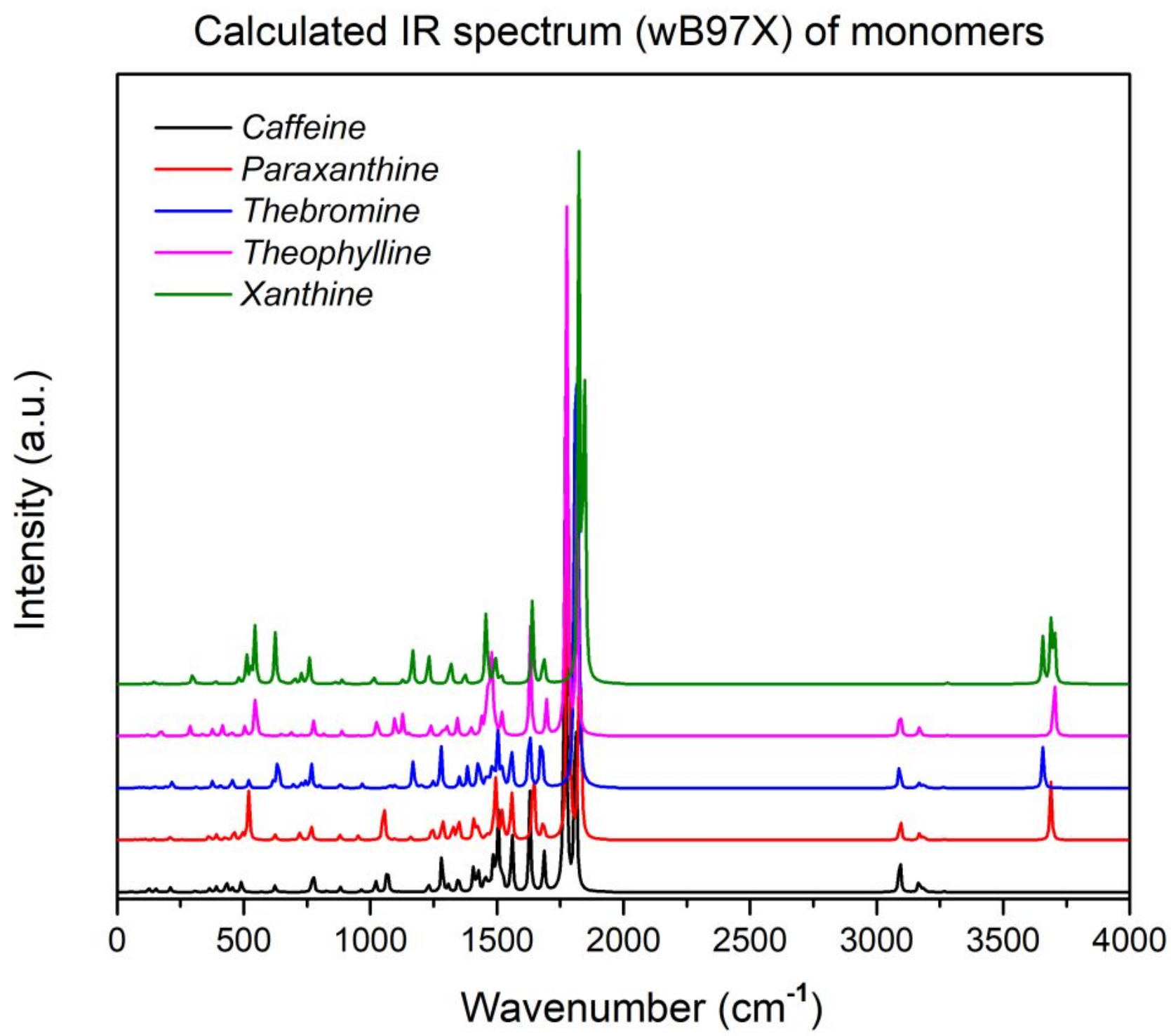

Figure S4. Theoretical IR spectra of caffeine (black), paraxanthine (red), theobromine (blue), theophylline (pink), and xanthine (green), from 0 cm^{-1} to 4000 cm^{-1} using DFT/ωB97X/6-311++g(d,p).

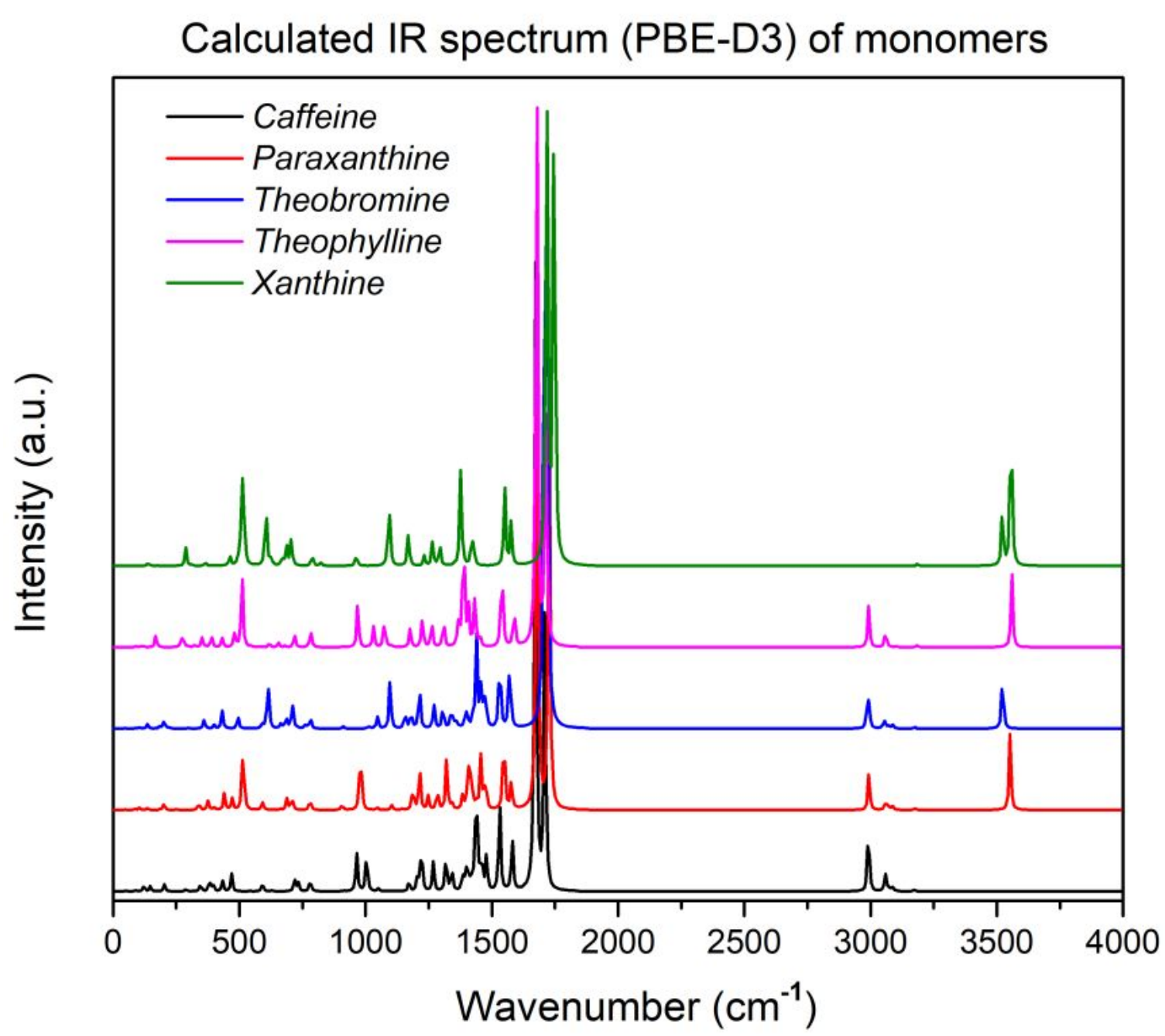

Figure S5. Theoretical IR spectra of caffeine (black), paraxanthine (red), theobromine (blue), theophylline (pink), and xanthine (green), from 0 cm^{-1} to 4000 cm^{-1} using DFT/PBE-D3/6-311++g(d,p).

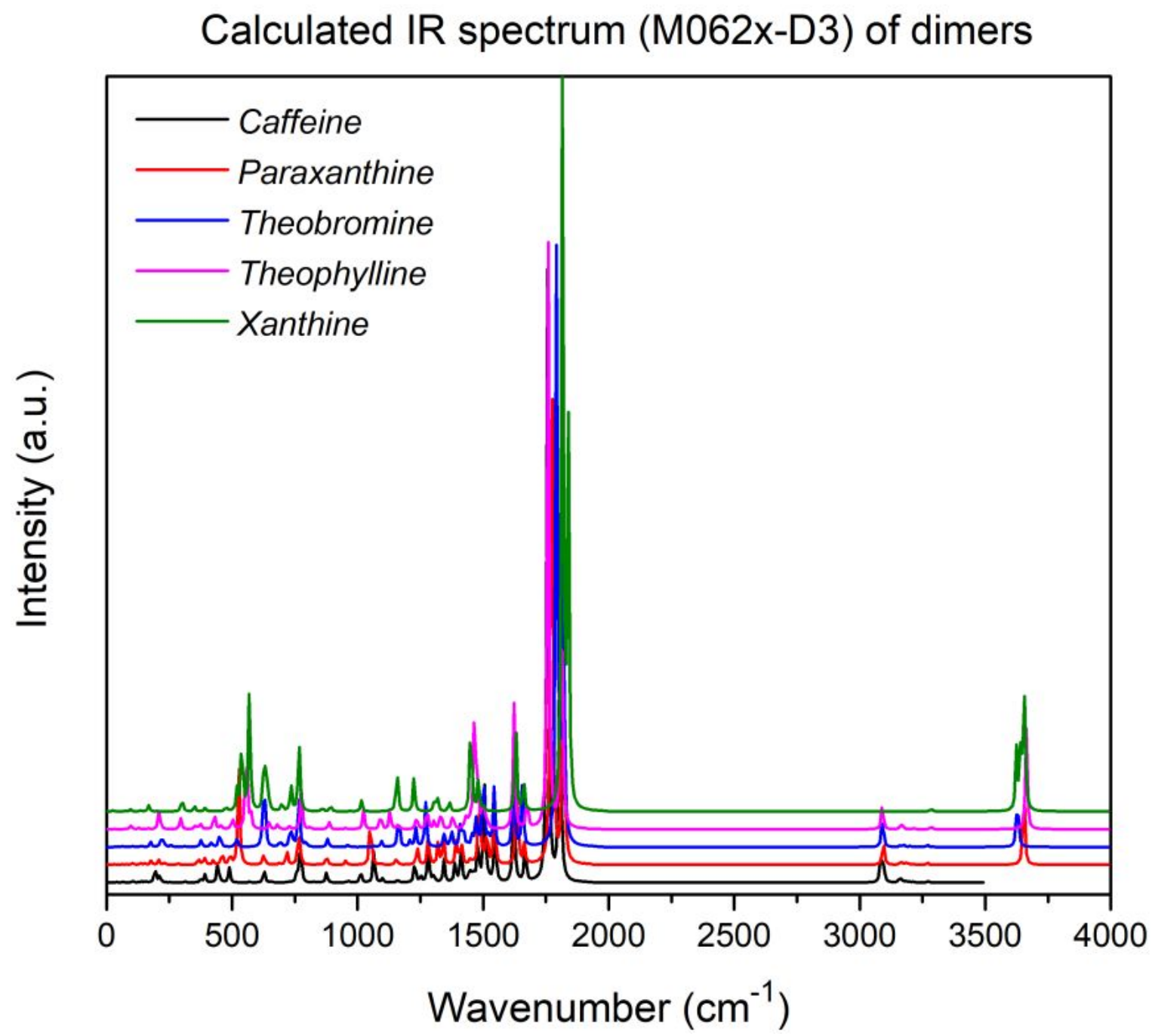

Figure S6. Theoretical IR spectra of caffeine (black), paraxanthine (red), theobromine (blue), theophylline (pink), and xanthine (green) dimers, from 0 cm^{-1} to 4000 cm^{-1} using DFT/M06-2X-D3/6-311++g(d,p).

MEP isosurface and Spatial distribution of the HOMO and LUMO orbitals through the Methylxanthines							
Monomers	MEP Isosurface	M06-2X		ωB97X		PBEPBE	
		HOMO	LUMO	HOMO	LUMO	HOMO	LUMO
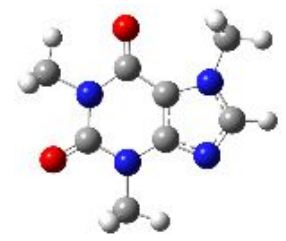 Caffeine	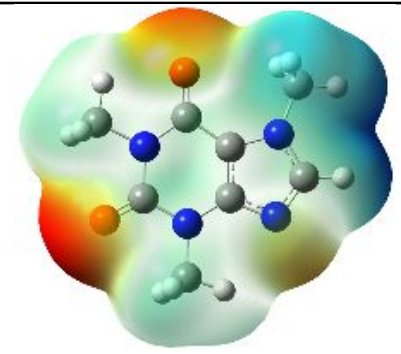	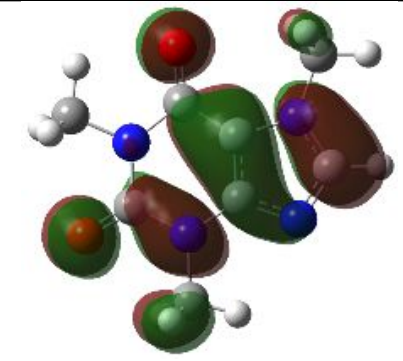	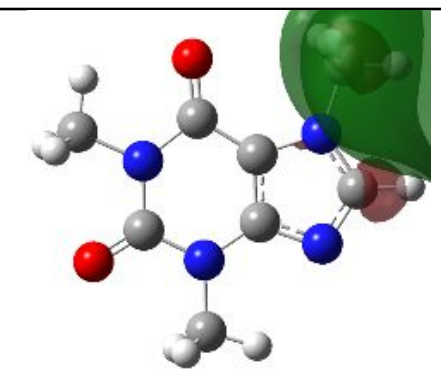	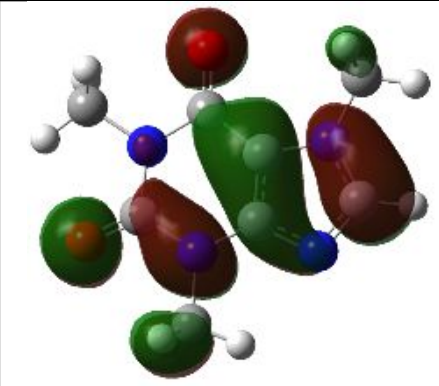	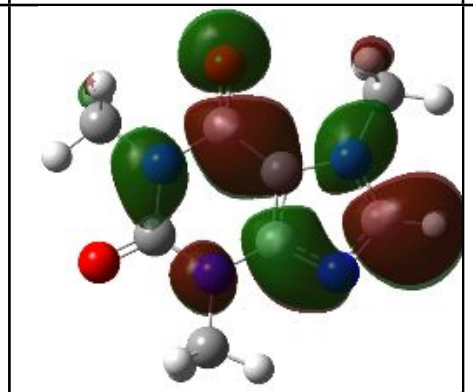	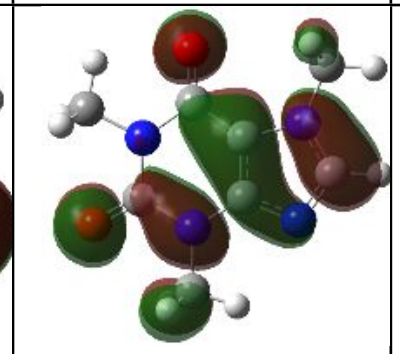	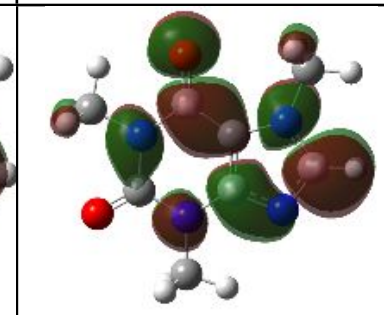
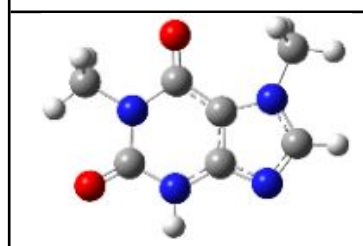 Paraxanthine	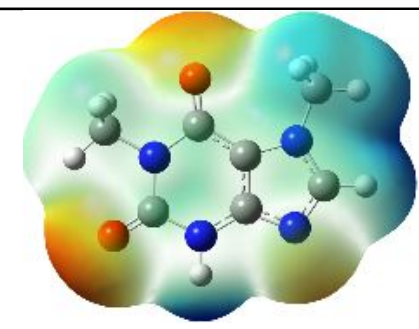	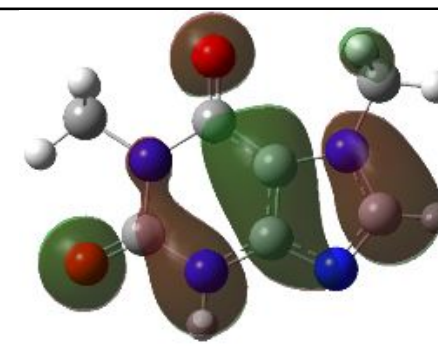	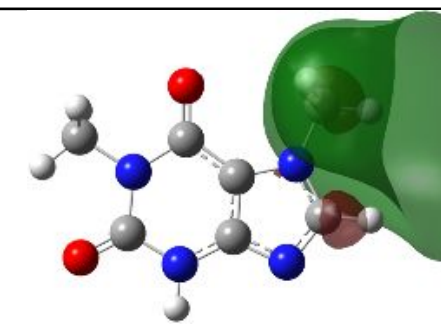	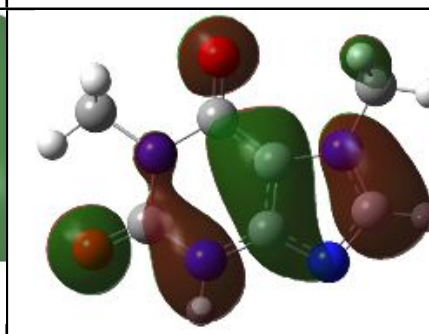	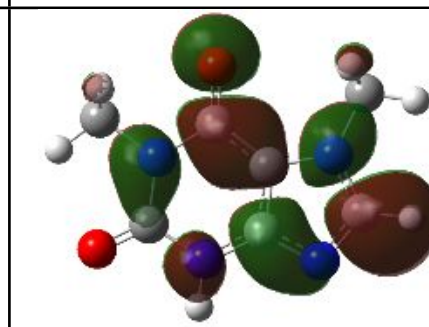	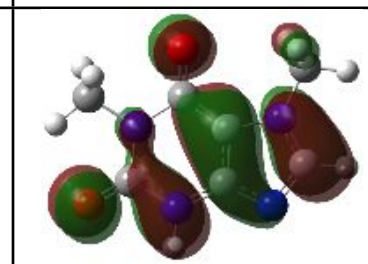	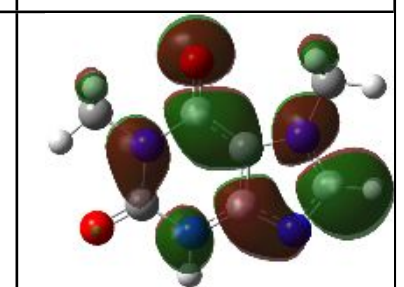
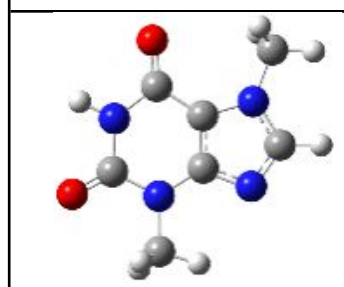 Theobromine	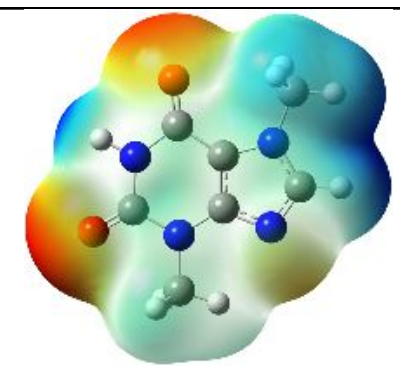	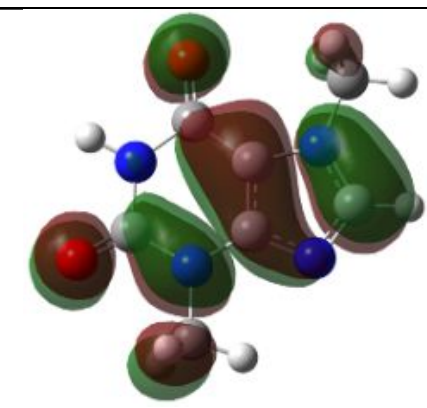	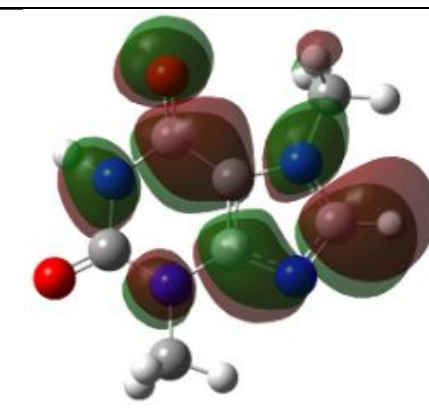	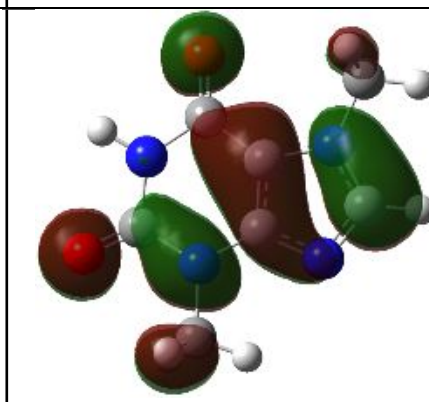	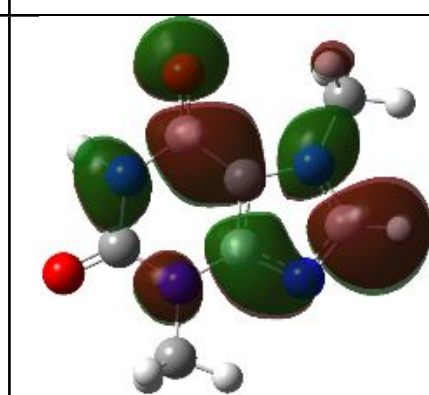	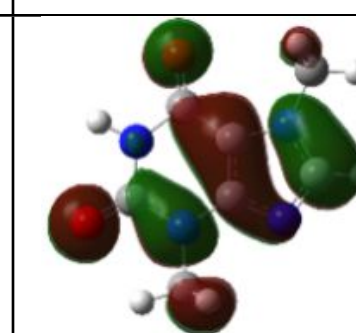	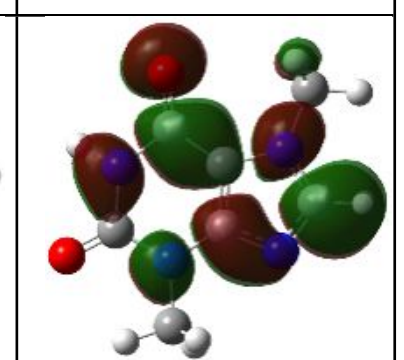
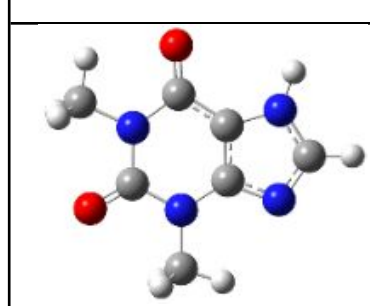 Theophylline	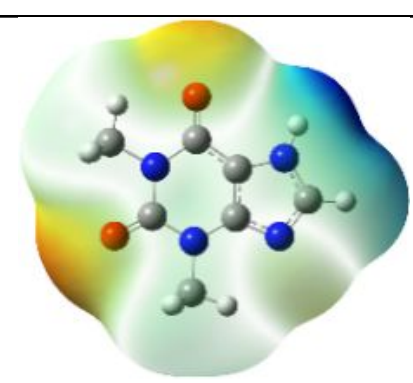	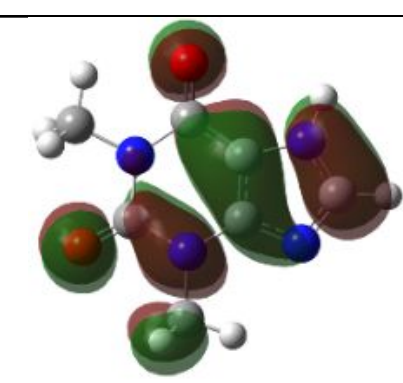	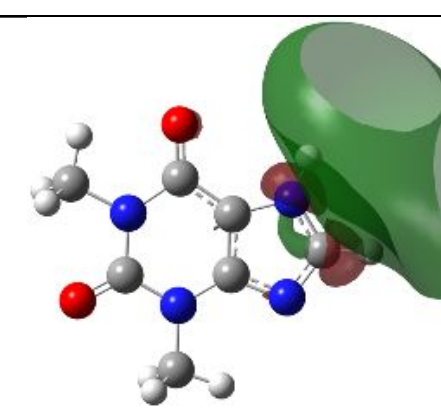	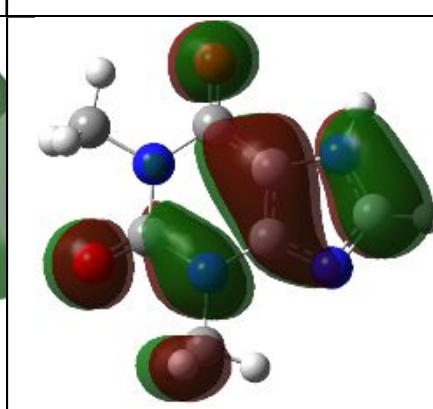	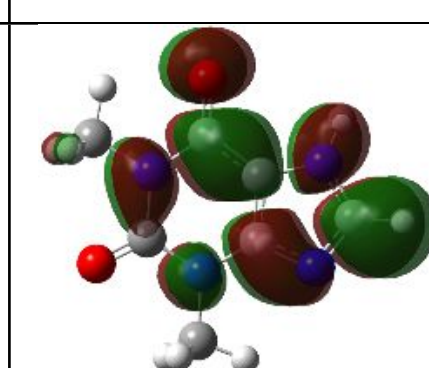	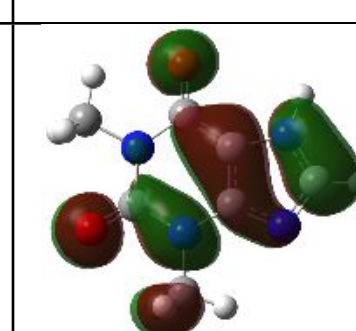	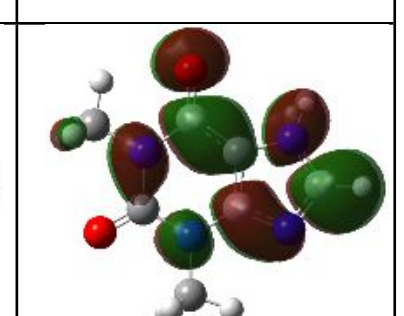

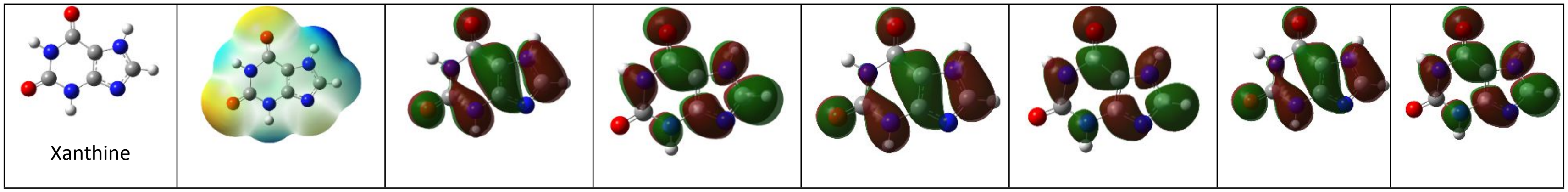

Figure S7. MEP isosurface and spatial distribution of the HOMO and LUMO orbitals (rendered at a constant isosurface value of 0.02 a.u.) through the Methylxanthines. Atom colors: red, oxygen; blue, nitrogen; gray, carbon; and white, hydrogen. Calculated at DFT/M06-2X/6-311++g(d,p), DFT/ωB97X/6-311++g(d,p), DFT/PBEPBE/6-311++g(d,p) respectively.

MEP isosurface and Spatial distribution of the HOMO and LUMO orbitals through the Methylxanthines Stacking Dimers							
Dimers	MEP Isosurface	M06-2X		PBEPBE		ωb97X	
		HOMO	LUMO	HOMO	LUMO	HOMO	LUMO
Caffeine 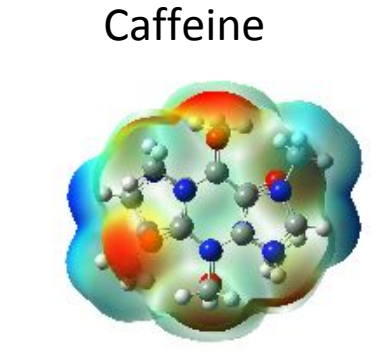	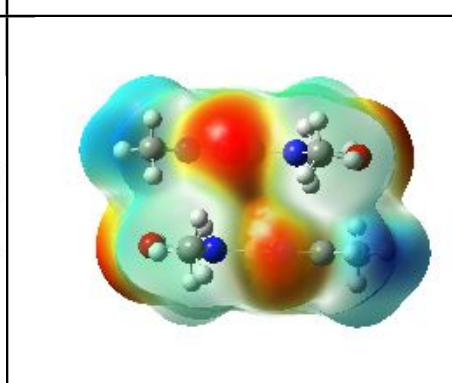	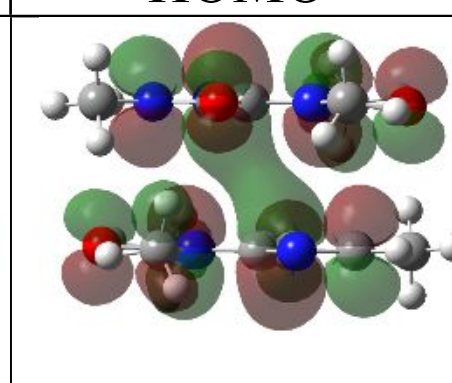	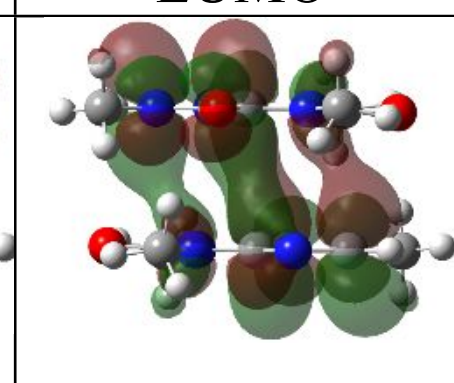	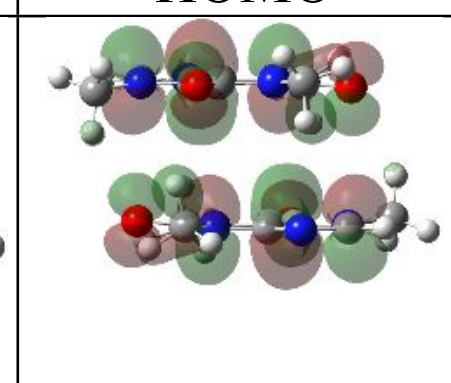	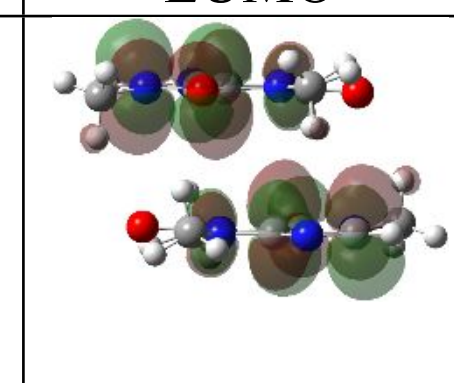	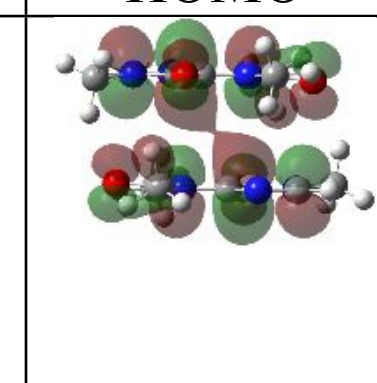	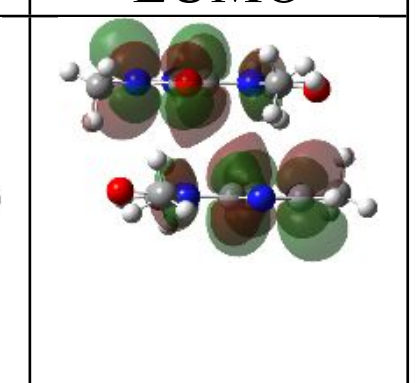
Paraxanthine 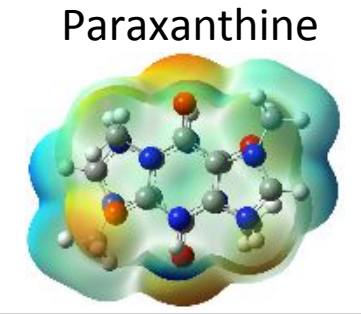	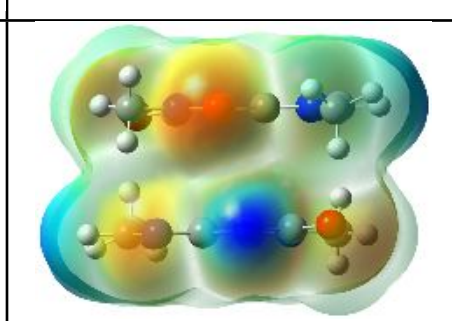	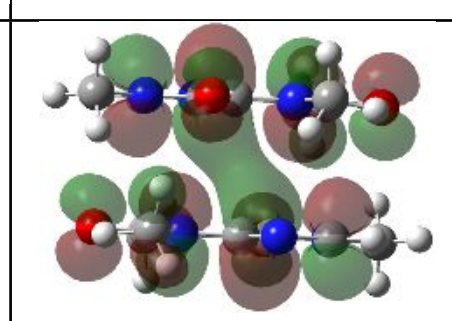	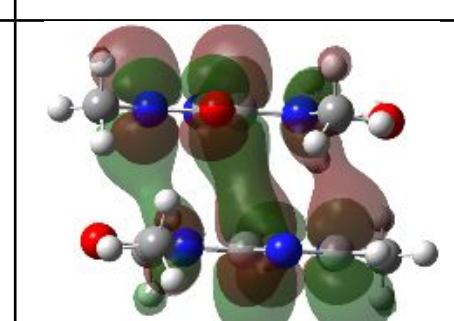	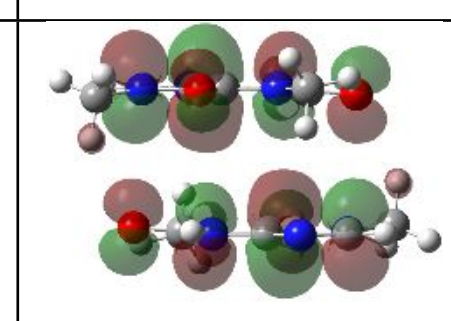	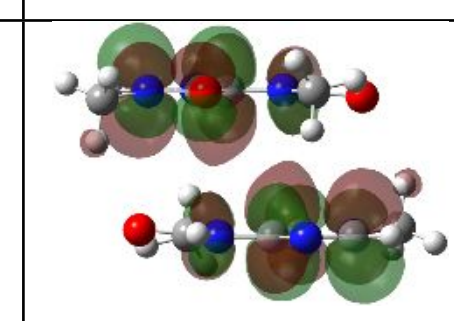	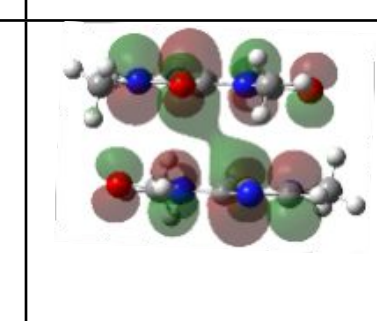	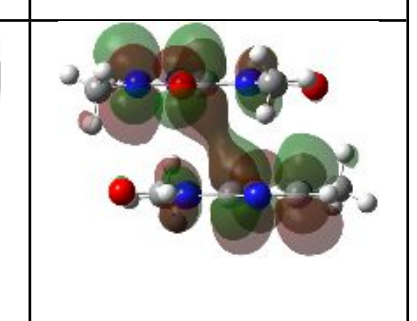
Theobromine 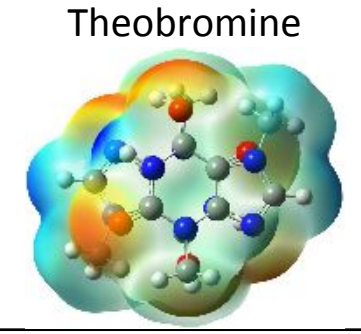	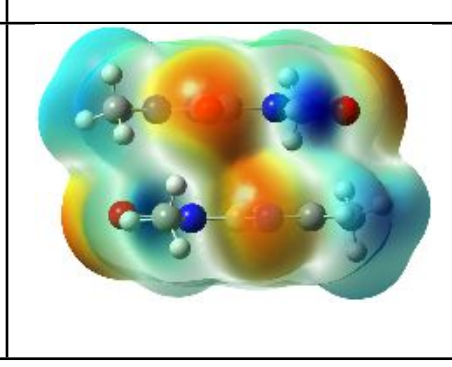	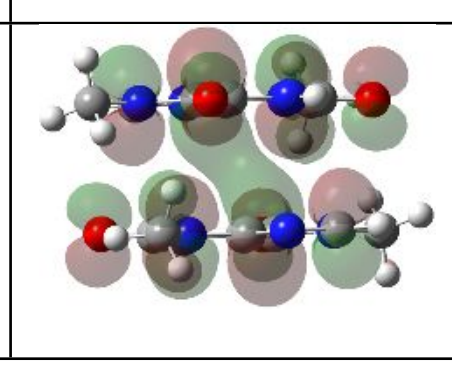	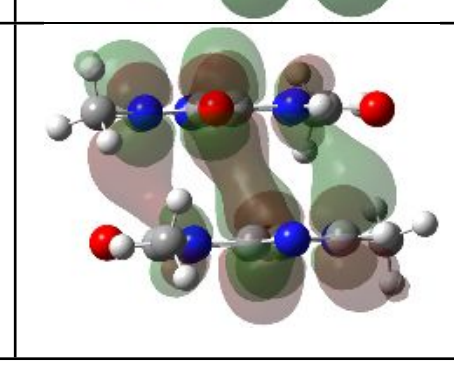	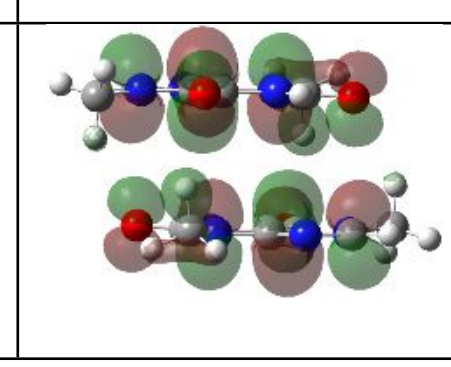	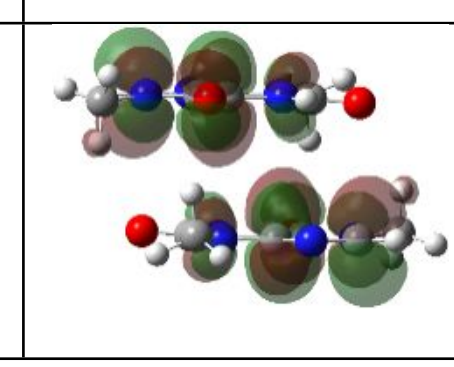	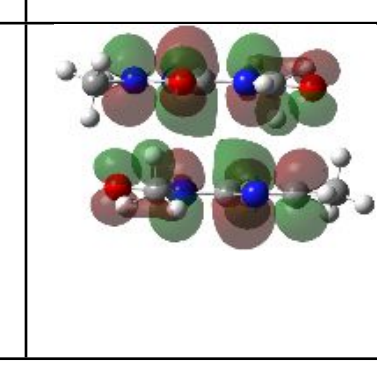	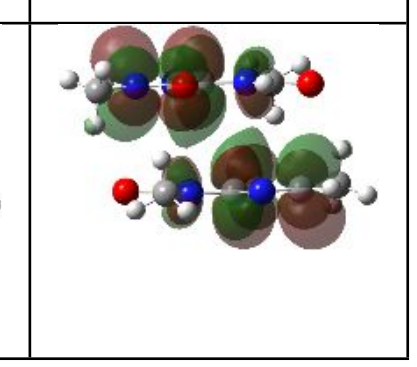

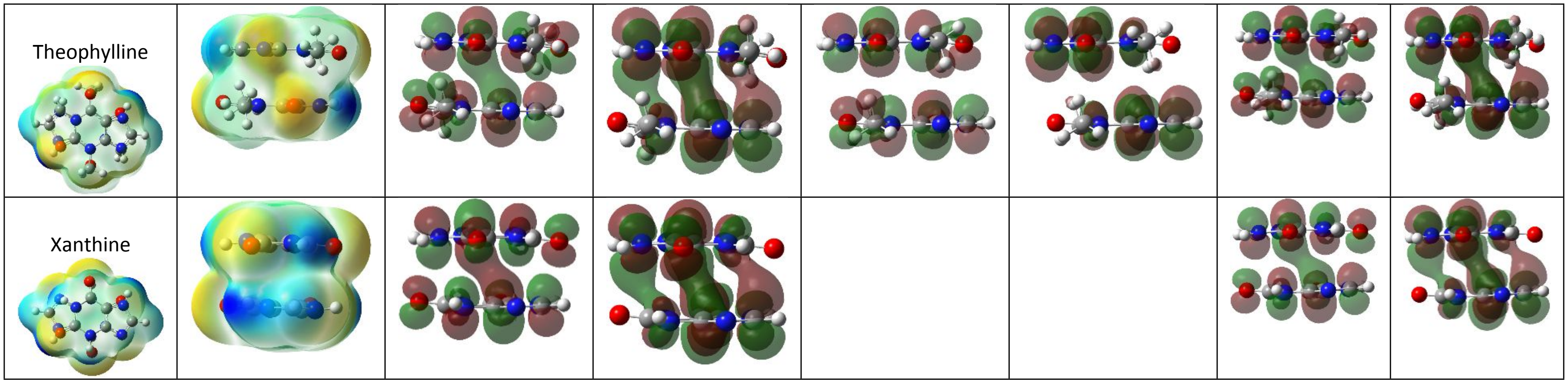

Figure S8. MEP isosurface and spatial distribution of the HOMO and LUMO orbitals (rendered at a constant isosurface value of 0.02 a.u.) through the Methylxanthines stacking dimers. Atom colors: red, oxygen; blue, nitrogen; gray, carbon; and white, hydrogen. Calculated at DFT/M06-2X/6-311++g(d,p), DFT/PBEPBE/6-311++g(d,p), DFT/ωB97X/6-311++g(d,p) respectively.

MEP isosurface and Spatial distribution of the HOMO and LUMO orbitals through the Methylxanthines Stacking Dimers							
Dimers	MEP Isosurface	M06-2X-D3		PBE-D3		wb97x	
		HOMO	LUMO	HOMO	LUMO	HOMO	LUMO
Caffeine 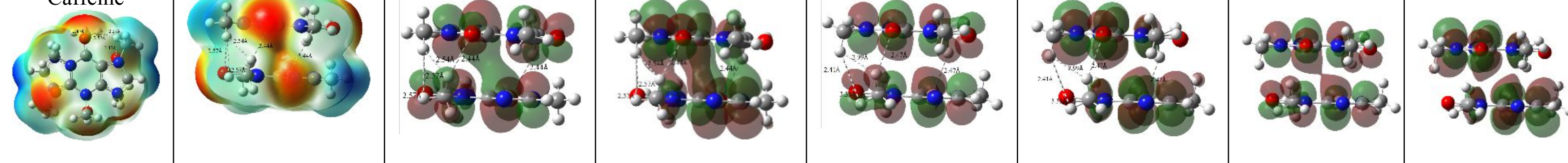							
Paraxanthine 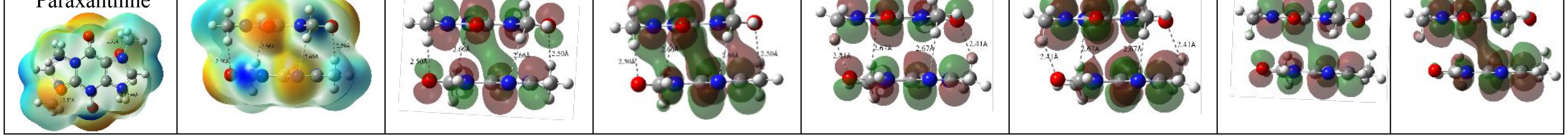							

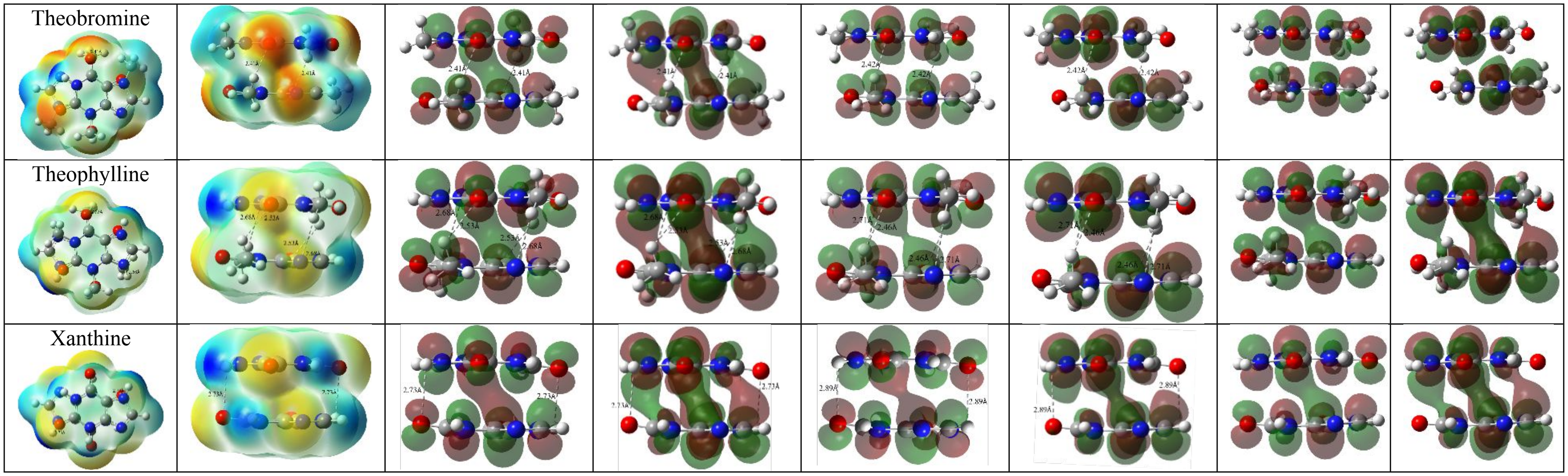

Figure S9. MEP isosurface and spatial distribution of the HOMO and LUMO orbitals (rendered at a constant isosurface value of 0.02 a.u.) through the Methylxanthines stacking dimers. Atom colors: red, oxygen; blue, nitrogen; gray, carbon; and white, hydrogen. Calculated at DFT/M06-2X-D3/6-311++g(d,p), DFT/PBE-D3/6-311++g(d,p), DFT/ωB97X/6-311++g(d,p) respectively.

Table S3. SAPT0 Energy Decomposition (kcal/mol) and relative contributions (%) for the five Methylxanthine dimers evaluated in this work, calculated using the M06-2X-D3, M06-2X, PBE-D3 and ωB97X functionals.

Dimer / Energy Component	Caffeine	Paraxanthine	Theobromine	Theophylline	Xanthine
**M06-2X-D3**					

Electrostatics (E_{elst})	-17.59 (35.7%)	-15.56 (36.5%)	-15.98 (36.7%)	-16.86 (38.2%)	-14.33 (40.9%)
Exchange(E_{exch})	31.96	27.73	27.94	28.24	22.12
Induction(E_{ind})	-3.73 (7.6%)	-2.96 (6.9%)	-3.46 (7.9%)	-3.13 (7.1%)	-2.45 (7.0%)
Dispersion(E_{disp})	-27.92 (56.7%)	-24.12 (56.6%)	-24.09 (55.4%)	-24.19 (54.7%)	-18.26 (52.1%)
**Total Attraction**	**-49.24 (100%)**	**-42.64 (100%)**	**-43.53 (100%)**	**-44.18 (100%)**	**-35.04 (100%)**
**Total Energy**	**-17.28**	**-14.91**	**-15.58**	**-15.94**	**-12.92**
**M06-2X**					
Electrostatics(E_{elst})	-17.74 (35.8%)	-15.70 (36.6%)	-16.13 (36.8%)	-16.97 (38.2%)	-14.49 (41.0%)
Exchange(E_{exch})	32.30	28.01	28.24	28.49	22.43
Induction(E_{ind})	-3.77 (7.6%)	-2.98 (6.9%)	-3.48 (7.9%)	-3.15 (7.1%)	-2.48 (7.0%)
Dispersion(E_{disp})	-28.06 (56.6%)	-24.24 (56.5%)	-24.21 (55.3%)	-24.29 (54.7%)	-18.39 (52.0%)

**Total Attraction**	**-49.57 (100%)**	**-42.92 (100%)**	**-43.82 (100%)**	**-44.41 (100%)**	**-35.36 (100%)**
**Total Energy**	**-17.27**	**-14.92**	**-15.58**	**-15.91**	**-12.92**
**PBE-D3**					
Electrostatics(E_{elst})	-14.83 (39.4%)	-12.91 (39.7%)	-14.23 (41.3%)	-12.42 (38.1%)	-10.33 (36.1%)
Exchange(E_{exch})	19.37	16.28	17.45	16.44	12.20
Induction(E_{ind})	-3.29 (8.7%)	-2.54 (7.8%)	-3.24 (9.4%)	-2.33 (7.2%)	-1.58 (5.5%)
Dispersion(E_{disp})	-19.56 (51.9%)	-17.06 (52.5%)	-17.03 (49.3%)	-17.82 (54.7%)	-13.73 (48.4%)
**Total Attraction**	**-37.68 (100%)**	**-32.51 (100%)**	**-34.50 (100%)**	**-32.57 (100%)**	**-25.64 (100%)**
**Total Energy**	**-18.31**	**-16.23**	**-17.04**	**-16.13**	**-13.45**
**ωB97X**					
Electrostatics(E_{elst})	-15.27 (38.9%)	-13.27 (39.4%)	-14.72 (41.1%)	-12.87 (37.8%)	-10.43 (39.5%)
Exchange(E_{exch})	20.96	17.51	18.91	18.01	13.36

Induction(E_{ind})	-3.31 (8.4%)	-2.60 (7.7%)	-3.30 (9.2%)	-2.38 (7.0%)	-1.63 (6.2%)
Dispersion(E_{disp})	-20.61 (52.7%)	-17.79 (52.9%)	-17.75 (49.7%)	-18.83 (55.2%)	-14.36 (54.3%)
**Total Attraction**	**-39.19 (100%)**	**-33.66 (100%)**	**-35.77 (100%)**	**-34.08 (100%)**	**-26.42 (100%)**
**Total Energy**	**-18.22**	**-16.15**	**-16.86**	**-16.07**	**-13.06**

E_{elst} (electrostatics), E_{exch} (exchange-repulsion), E_{ind} (induction), and E_{disp} (dispersion). Total Energy corresponds to the net SAPT0 interaction energy. Systems studied include caffeine, paraxanthine, theobromine, theophylline and xanthine dimer. All calculations were performed using the jun-cc-pVDZ basis set. The -D3 suffix denotes the inclusion of Grimme's empirical dispersion correction.

Table S4. Interaction Energy Components and Percentage of Dispersion Contribution for Caffeine and Xanthine Dimers Calculated via SAPT(DFT).

System / Functional	(E_{elst})	(E_{exch})	(E_{ind})	(E_{attr}) (Total)	% Dispersion	(E_{Total}) (SAPT)
Caffeine						
B97X	-14.74	-3.25	-16.33	-34.32	47.6%	-11.61
ωB97X-D	-14.20	-3.21	-16.44	-33.85	48.6%	-12.77
PBE-D3	-14.70	-3.13	-19.98	-37.81	52.8%	-13.77
Xanthine						
PBE-D3	-9.88	-1.50	-13.76	-25.14	54.7%	-10.42
ωB97X-D	-9.78	-0.98	-11.96	-22.72	52.6%	-8.56

E_{elst}, E_{ind}, and E_{disp} represent the electrostatic, induction, and dispersion components, respectively. E_{attr} is the sum of all attractive terms $(E_{elst} + E_{ind} + E_{disp})$. % Dispersion indicates the relative contribution of London dispersion to the total attractive force. Total SAPT energy includes the exchange-repulsion term (E_{exch}). Calculations were performed using the ωB97X, ωB97X-D, and PBE-D3 functionals as indicated.

Table S5. Optimized Total Energy (E_T), Energy of the Frontier Molecular Orbitals (HOMO and LUMO), Molecular Gap Energy (E_g), Global Molecular Descriptors (η, μ, ω,$\omega^{\pm}$, N'') and Cohesive Energy (E_{coh}) for the Methylxanthine monomers. [a]

	Methylxanthines				
Molecular Descriptor	Caffeine	Paraxanthine	Theobromine	Theophylline	Xanthine
E_T [b]	-18.504	-17.435	-17.435	-17.435	-15.297
E_{HOMO}	-7.5874	-7.8186	-7.7038	-7.7386	-8.1208
E_{LUMO}	-0.2937	-0.3242	-0.3353	-0.3593	-0.5233
E_g gap	7.2936	7.4944	7.3684	7.3793	7.5975
$I = -E_{HOMO}$	7.5874	7.8186	7.7038	7.7386	8.1208
$A = -E_{LUMO}$	0.2937	0.3242	0.3353	0.3593	0.5233
$\eta = (I - A)/2$	3.6468	3.7472	3.6842	3.6896	3.7987
$\mu = -(I + A)/2$	-3.9406	-4.0714	-4.0196	-4.0489	-4.3220
$\omega = \mu^2/2\eta$	2.1290	2.2118	2.1927	2.2216	2.4587
E_{coh}	-5.9683	-6.1384	-6.1406	-6.1382	-6.6851

[a] Optimization was performed via DFT/M06-2X/6-311++g(d,p). [b] Total energy is in units of keV; the rest of the parameters are given in eV.

Table S6. Optimized Total Energy (E_T), Energy of the Frontier Molecular Orbitals (HOMO and LUMO), Molecular Gap Energy (E_g), Global Molecular Descriptors (η, μ, ω,$\omega^{\pm}$, N'') and Cohesive Energy (E_{coh}) for the Methylxanthine monomers. [a]

	Methylxanthines				
Global Molecular Descriptors	Caffeine	Paraxanthine	Theobromine	Theophylline	Xanthine
E_T [b]	-18.506	-17.437	-17.437	-17.437	-15.299
E_{HOMO}	-8.6534	-8.8854	-8.7665	-8.8021	-9.1789
E_{LUMO}	1.006	0.9155	0.8905	0.9158	0.7033
E_g gap	9.6598	9.8009	9.6570	9.7180	9.8823
$I = -E_{HOMO}$	8.6534	8.8854	8.7665	8.8021	9.1789
$A = -E_{LUMO}$	-1.006	-0.9155	-0.8905	-0.9158	-0.7033
$\eta = (I - A)/2$	4.8299	4.9004	4.8285	4.8590	4.9411
$\mu = -(I + A)/2$	-3.8235	-3.9849	-3.9380	-3.9431	-4.2377
$\omega = \mu^2/2\eta$	1.5134	1.6202	1.6058	1.5999	1.8172
E_{coh}	-5.8767	-6.0450	-6.0473	-6.0449	-6.5865

[a] Optimization was performed via DFT/ωB97X/6-311++g(d,p). [b] Total energy is in units of keV; the rest of the parameters are given in eV.

Table S7. Optimized Total Energy (E_T), Energy of the Frontier Molecular Orbitals (HOMO and LUMO), Molecular Gap Energy (E_g), Global Molecular Descriptors (η, μ, ω,$\omega^{\pm}$, N'') and Cohesive Energy (E_{coh}) for the Methylxanthine monomers.[a]

	Methylxanthines				
Molecular Descriptor	Caffeine	Paraxanthine	Theobromine	Theophylline	Xanthine
E_T[b]	-18.490	-17.422	-17.422	-17.422	-15.286
E_{HOMO}	-5.4908	-5.6902	-5.6110	-5.6263	-5.9665
E_{LUMO}	-1.9926	-2.1001	-2.1379	-2.1161	-2.3604
E_g gap	3.4981	3.5901	3.4731	3.5101	3.6061
$I = -E_{HOMO}$	5.4908	5.6902	5.6110	5.6263	5.9665
$A = -E_{LUMO}$	1.9926	2.1001	2.1379	2.1161	2.3604
$\eta = (I - A)/2$	1.7490	1.7950	1.7365	1.7550	1.8030
$\mu = -(I + A)/2$	-3.7417	-3.8951	-3.8745	-3.8712	-4.1635
$\omega = \mu^2/2\eta$	4.0023	4.2261	4.3222	4.2694	4.8069
E_{coh}	-6.5392	-6.7255	-6.7277	-6.7254	-7.3242

[a] Optimization was performed via DFT/PBE/6-311++g(d,p). [b] Total energy is in units of keV; the rest of the parameters are given in eV.

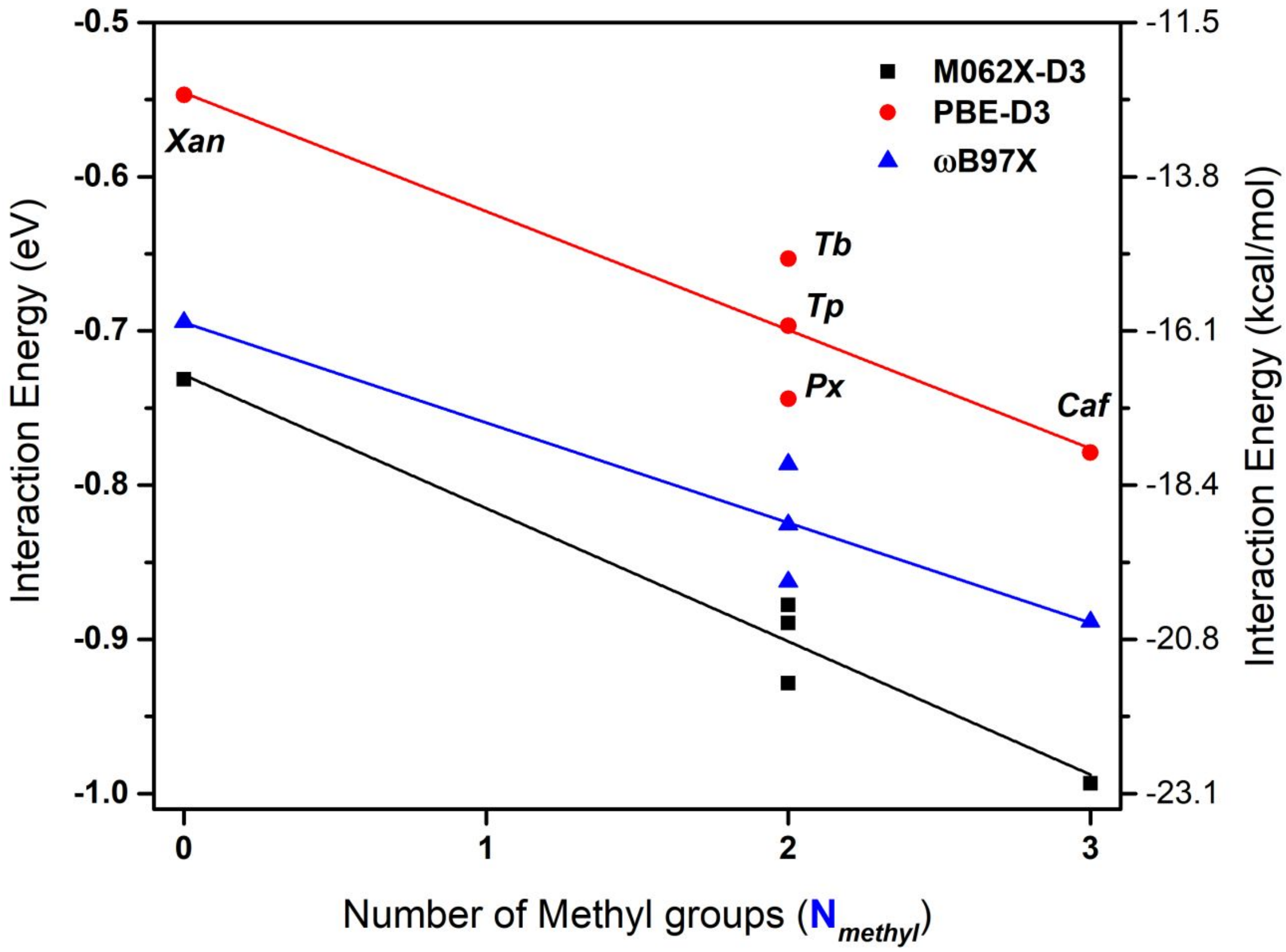

Figure S10. Interaction energy as a function of the number of methyl substituents per molecule of xanthine, calculated with ωB97X (blue triangles), PBE-D3 (red circles), and M06-2X-D3 (black squares) functionals with the 6-311++G(d,p) basis set. Xan = xanthine; Tb = theobromine; Tp = theophylline, Px = paraxanthine, Caf = caffeine. The solid lines represent linear fits to the data set.

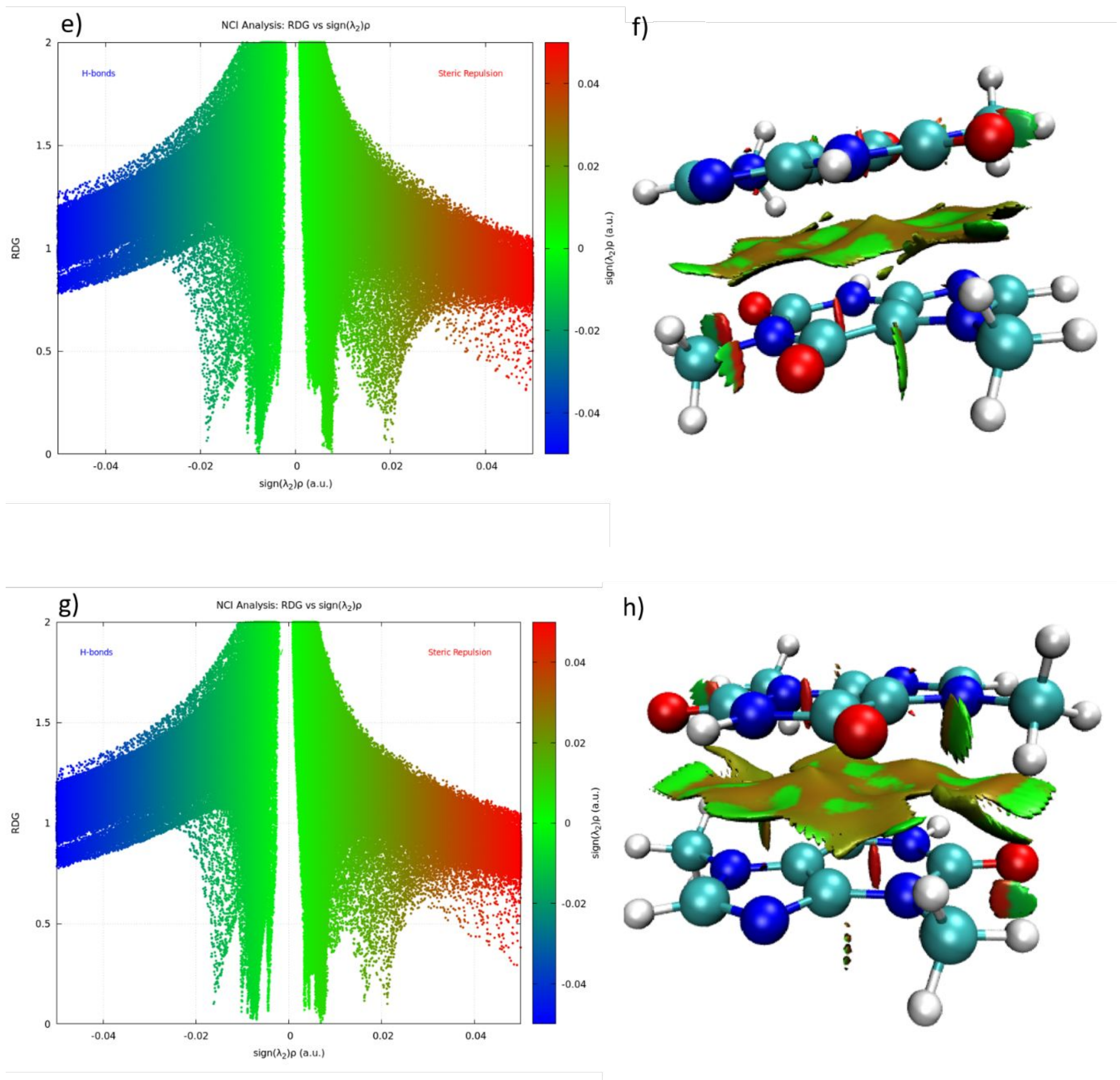

e)
NCI Analysis: RDG vs sign(λ2)ρ
H-bonds
Steric Repulsion
RDG
sign(λ2)ρ (a.u.)
f)
g)
NCI Analysis: RDG vs sign(λ2)ρ
H-bonds
Steric Repulsion
RDG
sign(λ2)ρ (a.u.)
h)

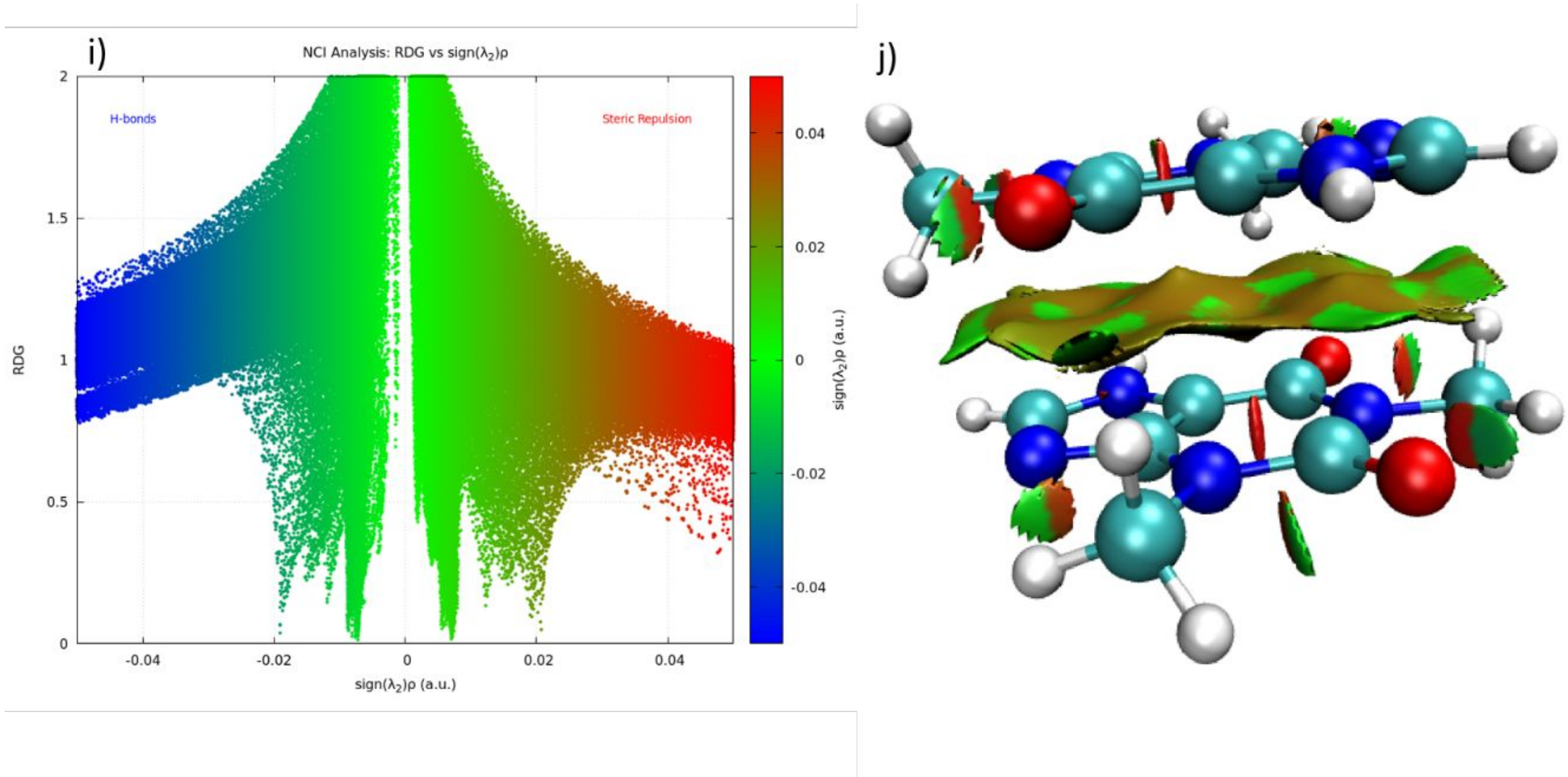

Figure S11. RDG vs *sign*$(\lambda_2)\rho$ plots and intermolecular interactions (the RDG isosurface is 0.5) of (e, f) paraxanthine, (g, h) theobromine, and (i, j) theophylline dimers. Color code for the isosurface: H–bonds (blue), vdW (green), steric (red).
